# Different Paths, Similar Pressures: Divergent Drivers of Genetic Diversity Despite Convergent Genomic Signatures of Selection in Response to Urban Intensity in Two Oligolectic Bee Species

**DOI:** 10.1111/mec.70370

**Published:** 2026-05-08

**Authors:** Lucie M. Baltz, Hanna Gardein, Henri Greil, Robert J. Paxton, Panagiotis Theodorou

**Affiliations:** ^1^ General Zoology, Institute for Biology Martin Luther University Halle‐Wittenberg Halle (Saale) Germany; ^2^ Institute for Bee Protection Julius Kühn Institute (JKI) – Federal Research Centre for Cultivated Plants Braunschweig Germany; ^3^ German Centre for Integrative Biodiversity Research (iDiv) Halle‐Jena‐Leipzig Leipzig Germany

**Keywords:** adaptive divergence, genotype by environmental association, Gradient forest, insect population genomics, latent factor mixed modelling, urban evolution

## Abstract

Urbanisation is a pervasive form of anthropogenic environmental change and a driver of contemporary evolution. Yet, it remains unclear how demographic processes and environmentally associated genomic variation shape genomic patterns in cities and whether these responses depend on species‐specific ecological traits. Here, we addressed this gap using whole‐genome sequencing of two related, diet‐specialised solitary bees ( *Andrena florea* and *Andrena vaga* ) that differ in dispersal‐related traits, rarity and host‐plant distribution, sampled along an urban intensity gradient. By integrating population and landscape genomic analyses, we quantified genetic diversity, demographic history, population structure and genotype–environment associations. Neutral genomic patterns differed strongly between species: *A. florea* showed lower genetic diversity, higher differentiation and a recent population decline, whereas *A. vaga* maintained higher diversity, connectivity and demographic stability. Genetic diversity was associated with species‐specific landscape features (edge density in *A. florea* and semi‐natural habitat in *A. vaga* ), rather than with urban intensity per se. Despite weak population structure, genotype–environment association analyses identified loci associated with urban intensity, and haplotype‐based scans detected genomic regions showing patterns consistent with positive selection. Functional annotation and cross‐species comparisons revealed partial convergence in candidate genes and functional pathways. Together, these results show that genomic responses to urbanisation cannot be explained by urban intensity alone, but instead emerge from the interaction between gene flow, genetic drift and selection, mediated by species‐specific ecological traits. This leads to divergent demographic trajectories but partly convergent genomic responses across species.

## 1 Introduction

Urbanisation reshapes landscapes, alters ecological connectivity and imposes novel environmental conditions on organisms worldwide (Li et al. 2022; McDonnell et al. 2009; Seto et al. 2012). By transforming continuous habitats into heterogeneous mosaics of built infrastructure, semi‐natural areas and managed green spaces, urban environments modify dispersal pathways, population connectivity and local population sizes (Munshi‐South and Richardson 2020). At the same time, urban environments expose organisms to novel selective pressures, including altered thermal regimes, pollution and chronic anthropogenic disturbance (Charmantier et al. 2024). These changes alter the interplay of gene flow, genetic drift and selection, with profound consequences for contemporary evolutionary dynamics and long‐term population persistence (Johnson and Munshi‐South 2017; Rivkin et al. 2019). A key challenge in urban evolutionary biology is therefore to understand how species persist in cities and which processes facilitate or constrain their existence—in particular, to what extent genomic patterns in urban landscapes reflect gene flow and genetic drift, environmentally associated selection, or their interaction (Johnson and Munshi‐South 2017; Rivkin et al. 2019; Szulkin et al. 2020). Disentangling these processes is essential for predicting which species can maintain viable populations in urban landscapes and how evolutionary responses may shape future biodiversity in cities.

Population genomic approaches provide a powerful framework to address this challenge because they allow the simultaneous inference of gene flow, genetic drift and selection across spatially complex landscapes (Szulkin et al. 2020). By enabling the joint analysis of these processes, such approaches have revealed contrasting genomic responses to urbanisation across taxa (Johnson and Munshi‐South 2017), ranging from reduced genetic diversity and increased population structure (e.g., Adducci et al. 2020; Braaker et al. 2017; Schmidt et al. 2020) to weak structure alongside signatures of selection (e.g., Babik et al. 2023; Bourgeois et al. 2025; Theodorou et al. 2018). These differences are generally attributed to variation in landscape permeability and species‐specific ecological traits, particularly dispersal ability and resource specialisation (Miles et al. 2019). In fragmented urban landscapes, species with limited movement capacity or specialised habitat requirements are expected to experience reduced connectivity and smaller effective population sizes, strengthening genetic drift and population structure (Johnson and Munshi‐South 2017; Rivkin et al. 2019; Szulkin et al. 2020). In contrast, more mobile or ecologically flexible taxa may maintain gene flow across urban landscapes, resulting in weak genome‐wide structure and the persistence of genetic diversity (Johnson and Munshi‐South 2017; Rivkin et al. 2019; Szulkin et al. 2020). Moreover, spatially consistent urban environmental gradients can promote environmentally structured genomic variation driven by selection, even in the presence of gene flow (Charmantier et al. 2024).

However, most studies focus on single species, making it difficult to disentangle whether observed genomic patterns reflect species‐specific demographic processes or shared responses to common urban environmental gradients, thereby limiting our ability to identify the ecological traits and processes that promote species persistence in urban landscapes. A powerful way to overcome this limitation is through comparative analyses of co‐occurring species that differ in key ecological traits. Bees are particularly well suited for such comparisons because their dispersal ability, resource use and nesting ecology directly influence gene flow, drift and exposure to environmental gradients (Hernandez and Suni 2024; López‐Uribe et al. 2019).

Here, we use a comparative population genomic approach to investigate how species‐specific ecological traits mediate genomic responses to urban landscape heterogeneity in two closely related solitary bees, *Andrena florea* and *Andrena vaga* . Although both species share similar life‐history characteristics (ground‐nesting, oligolectic, univoltine), they differ in key traits expected to influence gene flow and genetic drift. *Andrena florea* is smaller, less abundant and dependent on patchily distributed host plants, which may limit dispersal, reduce connectivity and effective population size and increase the strength of genetic drift, whereas *A. vaga* exploits more widespread resources, potentially facilitating higher connectivity, larger effective population sizes and the maintenance of genetic diversity across the landscape. Based on these trait differences, we formulated four hypotheses. First (H1), we expected species‐specific ecological traits to shape neutral genomic patterns through their effects on connectivity and genetic drift. Specifically, we predicted reduced diversity, greater spatial structure and stronger signatures of population decline in *A. florea* compared to *A. vaga* . We further predicted that genetic diversity would be associated with species‐specific landscape features—reflecting each species' dependence on different plant resources and habitat configuration—rather than urban intensity alone. Second (H2), if urban landscapes impose consistent environmental gradients, we predicted that loci associated with urban intensity would exhibit genotype–environment associations in both species, even in the presence of gene flow. Third (H3), if these gradients impose spatially structured selection, we predicted that genomic regions associated with urban intensity would show signatures consistent with positive selection. Finally (H4), we predicted that while neutral demographic patterns would differ between species due to their ecological traits, environmentally associated genomic responses would show partial convergence, reflecting shared exposure to urban selective pressures. By integrating spatially explicit landscape data with whole‐genome analyses, this study shows how gene flow, genetic drift and selection jointly shape genomic patterns and influence the processes underlying species persistence in urban environments.

## 2 Material and Methods

### 2.1 Study Species

**FIGURE 1:**
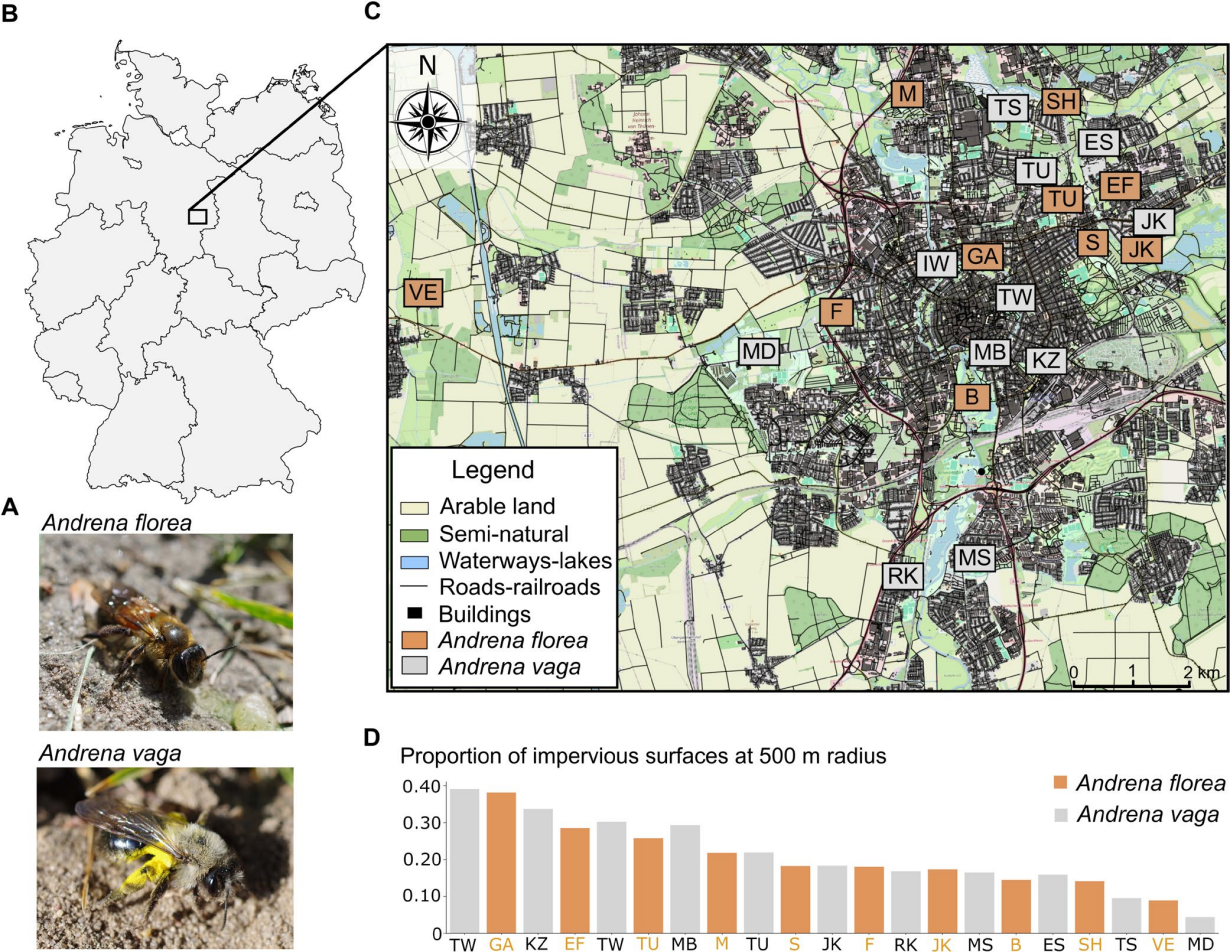
(A) *Andrena florea* and *Andrena vaga* (photographs by Henri Greil). (B) Map of Germany, where the black rectangle marks the city of Braunschweig. (C) Map of the city of Braunschweig, where the orange rectangles mark the 10 sites of *A. florea* and the grey dots mark the 11 sampling sites of *A. vaga* . (D) Proportion of impervious surfaces at 500 m radius around each site. The map was created with free vector map data licenced by OpenStreetMap Foundation (OSMF) under ODbL (https://opendatacommons.org/licenses/odbl/).

The bryony mining bee ( *Andrena florea* ; Figure 1A) is a mid‐sized (11–12 mm), solitary, ground‐nesting species and is common in southern and central Europe, including parts of Germany (Rasmont et al. 2013). It nests in shallow (≈10‐cm depth), compacted soil, often in small aggregations, and has a univoltine life cycle with activity from May to early August (Westrich 2019). *Andrena florea* is highly oligolectic, collecting pollen exclusively from plants of the genus *Bryonia* (Cucurbitaceae) in Germany; these occur patchily in vineyards, hedgerows and urban green spaces such as parks and gardens (Polidori and Federici 2019; Westrich 2019).

The grey‐backed mining bee ( *Andrena vaga* ; Figure 1A) is a larger (13–15 mm), solitary, ground‐nesting species, distributed across Europe and Asia (Rasmont et al. 2013). It forms large nesting aggregations, typically in sandy soils near river floodplains, with nests extending up to 60 cm deep (Gardein et al. 2025; Rezkova et al. 2012; Westrich 2019). Like *A. florea* , it is univoltine, with activity from March to May (Westrich 2019). *Andrena vaga* is oligolectic on *Salix* (willow), which is widespread along water bodies, riparian corridors and semi‐natural habitats across urban and rural landscapes (Bischoff et al. 2003; Westrich 2019).

### 2.2 Study Area and Sampling

Sampling was conducted during the flight period of both species in 2021 in and around the city of Braunschweig, Germany (Figure 1B,C; Tables S1 and S2). Braunschweig is located in Lower Saxony with a population of approximately 255,000 and spans 192 km^2^ (north–south: 19.1 km, west–east: 15.7 km) (Stadt Braunschweig 2025). Sampling strategies differed between species due to their ecology and feasibility. *Andrena florea* females were sampled using hand nets while foraging on their host plants (*Bryonia* spp.) in gardens and public spaces, as nests are difficult to locate. In contrast, *A. vaga* females were sampled at nesting aggregations in gardens, parks and other green spaces, as foraging individuals are difficult to access on *Salix* trees (Gardein et al. 2025). Our sampling strategy was therefore species‐specific but consistent within a species.

A total of 70 *A. florea* females were collected from 10 sites (7 individuals per site) and 114 *A. vaga* females were collected from 11 sites (10–12 individuals per site; Tables S1 and S2). Specimens were preserved in 100% ethanol and stored at −20°C. Sampling sites were distributed from the city centre to the outskirts and differed in impervious surface cover (Figure 1C,D; Tables S1 and S2). To ensure independence, sites were located at least 1 km apart (Gathmann and Tscharntke 2002; Greenleaf et al. 2007). This sampling design, spanning a gradient of urban intensity, provides the basis for evaluating neutral genomic patterns (H1) and environmentally associated genomic variation (H2–H4).

### 2.3 Local and Landscape Environmental Data

To investigate the environmental drivers of genome‐wide genetic diversity (H1), we quantified landscape variables known to influence bees (Baldock et al. 2019; Hopwood 2008; Theodorou, Radzevičiūtė, et al. 2020; Theodorou 2022). At each sampling location, landscape composition and configuration were quantified within a 500 m radius, reflecting typical foraging ranges of many bees, including *Andrena* species (Gathmann and Tscharntke 2002; Greenleaf et al. 2007), using QGIS v. 3.16.3. Land‐use data were obtained from Geofabrik (OpenStreetMap; https://download.geofabrik.de/) for the year 2020, the most recent dataset available prior to sampling. We quantified the following metrics: (i) the proportion of impervious surfaces (i.e., sealed surfaces), a widely used proxy for urban intensity in ecological and evolutionary studies (McDonnell and Hahs 2009; Niemelä et al. 2011), (ii) the proportion of urban green land uses (i.e., parks, allotment gardens, recreation grounds, cemeteries), (iii) the proportion of semi‐natural cover (i.e., meadows and shrubs) and (iv) edge density, defined as the total length (m) of all patch edges divided by the total area (m^2^), used here as a proxy for ecotone availability (Tables S1 and S2). For *A. vaga* , we additionally quantified water‐body edge length as a proxy for *Salix* resource availability, derived from high‐resolution land‐use maps provided by the Niedersächsisches Ministerium für Umwelt, Energie und Klimaschutz (Table S2). Because individuals were collected from nesting aggregations, aggregation size was also estimated by measuring aggregation area and nest density across four 1 m^2^ plots per site—two with highest visible nest density and two in more sparsely occupied areas—which were averaged and then multiplied by the total aggregation area to obtain a relative aggregation size metric (Table S2).

### 2.4 DNA Extraction, Whole Genome Sequencing and SNP Genotyping

Genomic DNA was isolated from thoracic muscle tissue using a CTAB‐based protocol with proteinase K digestion, followed by chloroform:isoamyl alcohol purification. DNA concentration and integrity were assessed with a Qubit 3.0 fluorometer (Thermo Fisher Scientific, Waltham, MA, USA) and an Epoch spectrophotometer (BioTek, Winooski, USA). Samples were standardised to 15 ng/μL prior to library preparation. DNA library preparation was performed using the Illumina DNA Prep kit, followed by whole‐genome sequencing (approximately 6 Gbp per sample) on a NovaSeq 6000 S4 flow cell with 150‐bp paired‐end reads, conducted by SNPsaurus (Eugene, OR, USA). The number of read pairs was high and consistent across species and populations ( *A. florea* : mean = 19,477,022; SD = 2,969,268; *A. vaga* : mean = 19,267,947; SD = 3,179,331; Tables S4 and S5).

Raw reads were quality‐checked with FastQC v. 0.12.0 (Andrews 2010) and trimmed using BBDuk (BBMap v. 38.91) with parameters set to ktrim = *r*, *k* = 17, mink = 8, hdist = 1, tpe, tbo, qtrim = rl, trimq = 10. Species‐specific draft reference genomes for *A. florea* and *A. vaga* were generated to enable accurate read alignment and variant calling (see Methods S1 for assembly details; Table S3). Cleaned reads were mapped to the respective reference genomes ( *A. florea* : GCA_052575755.1; *A. vaga* : GCA_052575775.1) using bwa‐mem2 v. 2.2.1 (Vasimuddin et al. 2019). PCR duplicates were marked using GATK v. 4.2.2.0 (Van der Auwera et al. 2013). Sequencing depth and coverage were analysed using SAMtools v. 1.7 (Li et al. 2009) with the commands samtools ‐‐depth and depth ‐a. Sequencing depth and genome coverage were high and consistent across species and populations (Sequencing depth: *A. florea* : mean = 15.04, SD = 6.64; *A. vaga* : mean = 18.66, SD = 6.54; Genome coverage: *A. florea* : mean = 99%, SD = 0; *A. vaga* : mean = 98.61%, SD = 1.36; Tables S4 and S5).

Single‐nucleotide polymorphism (SNP) calling was performed using the CallVariants module of BBMap v. 38.91, with the following parameters: ploidy = 2, ow = t, nopassdot = f, minad = 2, minavgmapq = 15, minreadmapq = 15 and strandedcov = t. This initial dataset consisted of 4,573,141 SNPs for *A. florea* and 3,882,609 SNPs for *A. vaga* . Subsequent variant filtering was conducted using VCFtools v. 0.1.16 (Danecek et al. 2011). Initial filtering retained SNPs genotyped in at least 50% of individuals, Phred quality ≥ 30, minor allele count ≥ 3 and a minimum per‐individual genotype depth of 3 reads. Genotyping error rates associated with low sequencing depth (≤ 5×) were estimated using the ErrorCount.sh script from the dDocent pipeline (Puritz et al. 2014). This script estimates whether the variant caller may be introducing genotyping errors due to low read depth by calculating expected error rates based on binomial probability. Estimated error rates were minimal, ranging from 0.008% to 0.03% for *A. florea* and 0.005% to 0.02% for *A. vaga* .

For genetic diversity, divergence, population structure and demographic analyses, additional filtering was applied to retain high‐confidence loci using the following thresholds: ‐‐max‐missing 0.95, ‐‐maf 0.05, ‐‐min‐meanDP 5, ‐‐max‐meanDP 100 and ‐‐remove‐indels. Individuals with > 50% missing data were removed using VCFtools. To exclude potential paralogous loci, SNPs with an observed heterozygosity > 0.6 were filtered out using VCFtools (Jackson et al. 2018; Taylor et al. 2014). Additionally, to mitigate biases due to linkage disequilibrium (LD), SNP pruning was performed using PLINK v. 1.9 (Purcell et al. 2007) with the command ‐‐indep 50 5 0.5, removing SNPs with a pairwise correlation > 0.5 within a 50‐SNP window. Because sampling from nesting aggregations or flower patches may increase the likelihood of collecting related individuals—particularly in *A. vaga* collected from the same nesting aggregation—we explicitly assessed relatedness among sampled individuals prior to downstream analyses. Pairwise relatedness was estimated with VCFtools ‐‐relatedness2 (Manichaikul et al. 2010). No highly related individuals were detected in either species. The final dataset comprised 70 individuals and 222,251 SNPs for *A. florea* and 114 individuals and 243,751 SNPs for *A. vaga* .

For genotype‐environment association (GEA) and selective sweep analyses, we applied a more relaxed filtering approach using the thresholds: ‐‐max‐missing 0.75, ‐‐maf 0.05, ‐‐min‐meanDP 5, ‐‐max‐meanDP 100 and ‐‐remove‐indels. Individuals with > 50% missing data were removed and paralogous loci were excluded. No LD pruning was applied. These filtering parameters were chosen based on best practice for next‐generation sequencing data processing (Hemstrom et al. 2024). After filtering, the final dataset comprised 70 individuals and 1,180,945 SNPs for *A. florea* and 114 individuals and 558,887 SNPs for *A. vaga* .

### 2.5 Genetic Diversity, Divergence, Population Structure and Demographic Trends (H1)

To test H1, that species‐specific ecological traits shape neutral genomic patterns through their effects on connectivity and genetic drift, we quantified genome‐wide genetic diversity, population differentiation and genetic structure. Genome‐wide genetic diversity, measured as expected heterozygosity (*H*_EXP_), was calculated for each sampling location using the R package *dartR* v. 2.9.7 (Gruber et al. 2018). To obtain adjusted genome‐wide values, we quantified the number of callable sites per population from an all‐sites VCF file generated with bcftools mpileup and bcftools call ‐A. Adjusted genome‐wide heterozygosity (*adjH*_EXP_; Schmidt et al. 2021) was calculated as:

$${\text{adjH}}_{\mathrm{EXP}}=H_{\mathrm{EXP}}\times \frac{\text{number of polymorphic loci}}{\text{number of callable sites}}$$

(Gruber et al. 2018). *H*_EXP_ and the *adjH*_EXP_ were highly correlated within species (Pearson's correlation: *A. florea* , *r* = 0.99; *p* < 0.001; *A. vaga* , *r* = 0.92; *p* < 0.001). Genetic diversity (*adjH*_EXP_) was compared between species using a linear model (LM), including sample size per population as a covariate.

Pairwise *F*_ST_ values were estimated to examine genetic divergence among populations using the R package *StAMPP* v. 1.6.3 (Pembleton et al. 2013), with confidence intervals obtained from 1000 bootstrap replicates. The relationship between genetic and geographic distance (isolation by distance, IBD) was evaluated using Mantel tests in the R package *ade4* v. 1.7–22 (Dray and Dufour 2007), employing linearised *F*_ST_ values (*F*_ST_/(1−*F*_ST_)) and log‐transformed geographic distances. Genetic differentiation (*F*_ST_) was compared between species using a weighted least squares model, with geographic distance included as a covariate.

Population structure was inferred using the *find.clusters* function implemented in the *adegenet* R package (Jombart et al. 2008; Jombart and Ahmed 2011), with the number of clusters determined using the Bayesian Information Criterion (BIC). Values of *K* from 1 to 11 ( *A. florea* ) and 1 to 12 ( *A. vaga* ) were tested. Population structure was further visualised using principal coordinates analysis (PCoA) implemented in *dartR* (Gruber et al. 2018).

To assess the effects of landscape composition and configuration on genetic diversity, we fitted separate LMs for each species. To specifically examine the total impact of urban intensity, we first fitted models using impervious surface cover as a single predictor. We then evaluated additional landscape variables, including urban green space, semi‐natural cover and edge density. For *A. vaga* , water‐body edge length and aggregation size were also included as predictors. Multicollinearity among predictors was evaluated using variance inflation factors (VIF), with a threshold of five (Menard 2002). All models met this criterion (VIF < 5), indicating that multicollinearity was not an issue. Additionally, spatial autocorrelation was tested using Moran's *I*, implemented in the *ape* R package (Paradis and Schliep 2019). No significant spatial autocorrelation was detected (*p* > 0.05), ensuring that spatial dependence did not bias model outputs. Given the large number of predictors and the risk of overfitting, model selection was performed using the *dredge* function from the *MuMIn* package (Barton 2020). For each species, global models were fitted and ranked using an automated model selection procedure based on the Akaike Information Criterion (AIC), with a maximum of two predictors per model. Models with ΔAIC < 2 were subsequently averaged to obtain model‐averaged parameter estimates. All statistical analyses were performed using the statistical software R v. 4.5.0 (R Core Team 2025).

To further evaluate H1, we reconstructed recent effective population size (*Ne*) trajectories to assess differences in demographic history between species. *Ne* trends were inferred from genome‐wide SNP data using GONE (Santiago et al. 2020). Due to weak population structure, individuals were grouped into a single metapopulation per species. To minimise the influence of selection, SNPs associated with urban intensity (LFMM + GF; see Section 2.6) and additional outlier loci identified using pcadapt v. 4.3.5 (Luu et al. 2017) were excluded (FDR < 0.10). GONE analyses were performed using 199,848 SNPs for *A. florea* and 235,776 SNPs for *A. vaga* . Contigs with fewer than 100 SNPs were excluded. GONE was run with default parameters, assuming a recombination rate of 8.7 cM/Mb based on *Bombus terrestris* (Liu et al. 2017). Estimates beyond 250 generations and within the first five generations were excluded due to reduced reliability (Nadachowska‐Brzyska et al. 2022; Santiago et al. 2020). Because LD‐based methods can substantially underestimate absolute *Ne* in haplodiploid species (Wang et al. 2016), and recombination rates may differ between Andrenidae and Apidae, absolute *N*e estimates should be interpreted cautiously. To aid interpretation, all *N*e estimates were log‐transformed prior to visualisation.

### 2.6 Genotype–Environment Associations and Haplotype‐Based Selection Scans (H2–H3)

To test H2, in which we predicted genotype–environment associations (GEA) with urban intensity, we identified SNPs whose allele frequencies were associated with impervious surface cover using two complementary GEA methods: latent factor mixed models (LFMM) (Caye et al. 2019; Frichot et al. 2013) and Gradient forest (GF) (Ellis et al. 2012). Missing genotypes were imputed using the most common genotype at each SNP, and analyses were conducted using population‐level allele frequencies. LFMM analyses were performed using ridge regression in the *lfmm* R package (Caye et al. 2019), with the number of latent factors determined from population structure analyses (*K* = 1). An alternative model (*K* = 2) for *A. florea* yielded consistent results. To control for multiple testing, empirical significance thresholds were derived from 1000 permutations of the environmental variable, generating a null distribution of *p*‐values. SNPs with *p*‐values below the 0.1st percentile of this distribution were considered significantly associated with urban intensity (*p* < 2.51 × 10^−7^ for *A. florea* , *p* < 1.63 × 10^−6^ for *A. vaga* ). Gradient forest analyses were conducted using the *gradientForest* R package (Ellis et al. 2012), with allele frequencies as response variables and impervious surface cover as the predictor. Models were run with 500 trees and candidate SNPs were defined as those within the top 1% of cumulative importance values. Urban intensity‐associated SNPs identified by both methods (LFMM ∩ GF) were considered robust candidates.

To test H3, we used the haplotype‐based statistic nSL (Ferrer‐Admetlla et al. 2014) to identify genomic regions showing signatures of positive selection associated with urban intensity. We calculated nSL using *selscan* v. 2.1.1 (Szpiech and Hernandez 2014) on the dataset used for GEA analyses, treating each species as a single metapopulation given the weak population structure. Because raw nSL values depend on allele frequency, scores were normalised genome‐wide using the *norm* utility distributed with *selscan*, applying 100 derived allele frequency bins. This procedure yields standardised nSL values (*Z*‐scores) that are comparable across loci and contigs while reducing biases associated with allele‐frequency heterogeneity. Candidate loci were defined as SNPs with |nSL| ≥ 2. Adjacent outlier SNPs within 50 kb were merged into candidate sweep regions.

To evaluate the relationship between selective sweeps and environmentally associated genomic variation, nSL outlier regions were compared with GEA results (LFMM ∩ GF). Overlap was defined as SNPs located within ±10 kb of sweep regions, allowing for linkage disequilibrium extending beyond detected sweep intervals (Beye et al. 2006; Stolle et al. 2011). Significance was evaluated using permutation tests (1000 permutations) in which SNP positions were randomly reassigned within contigs while preserving SNP density.

To interpret the biological relevance of loci associated with urban intensity, we functionally characterised candidate regions identified by both GEA methods (LFMM ∩ GF). Additional analyses restricted to loci identified by all methods (LFMM ∩ GF ∩ nSL) yielded similar results and are reported in the Supporting Information (Tables S14 and S15). For each species, genomic regions spanning ±10 kb around candidate SNPs were extracted and aligned to the *Apis mellifera* reference genome (Amel_HAv3.1) using BLASTn (*E*‐value < 1e−10) (Altschul et al. 1990). Genes located within these regions were considered putative targets of selection. Gene Ontology (GO) annotations were obtained from the Hymenoptera Genome Database (https://hymenoptera.elsiklab.missouri.edu).

Functional enrichment was assessed using GO term overrepresentation tests with the *topGO* R package v. 2.54.0 (Alexa and Rahnenfuhrer 2023), using *A. mellifera* as the reference background, as no annotated genome is currently available for either study species. We employed the conservative *elim* algorithm, which reduces false positives by removing genes from ancestor GO terms once they are associated with significant child terms. As the tests performed by this algorithm are not independent, multiple testing correction is not directly applicable and therefore raw *p*‐values < 0.05 were considered indicative of significant enrichment (Alexa and Rahnenfuhrer 2023). A minimum node size of 5 annotated genes was used to filter out GO terms with extremely low support (Alexa and Rahnenfuhrer 2023). The top 10 GO terms for each ontology (biological process, molecular function, cellular component) were retained for visualisation.

### 2.7 Cross‐Species Functional Convergence in Environmentally Associated Genomic Responses (H4)

To test H4, in which we predicted partial convergence in environmentally associated genomic responses across species, we assessed overlap and functional similarity of candidate loci between species. Candidate orthologue regions were identified by aligning genomic sequences surrounding urban intensity‐associated SNPs (LFMM ∩ GF) between *A. florea* and *A. vaga* using BLASTn (*E*‐value < 1e−10) (Altschul et al. 1990). The significance of overlap was evaluated using hypergeometric tests via the *phyper* function in R, considering only shared genomic regions between species. GO enrichment tests for the overlapping genes were conducted using the R package *topGO* v. 2.54.0 (Alexa and Rahnenfuhrer 2023), and the top 10 GO terms from each ontology were retained for visualisation.

Functional convergence was further quantified by calculating semantic similarity among enriched GO terms using the R package *GOSemSim* (Yu 2020; Yu et al. 2010) and the Wang method. Analyses were restricted to a shared GO universe to ensure comparability. Pairwise similarity was calculated using the best‐match average (BMA) approach, and ontology‐specific values were averaged to obtain an overall similarity score. Significance was assessed using permutation tests (10,000 permutations), comparing observed similarity to a null distribution generated by random sampling of GO terms.

## 3 Results

### 3.1 Genetic Diversity, Divergence, Population Structure and Demographic Trends (H1)

**FIGURE 2:**
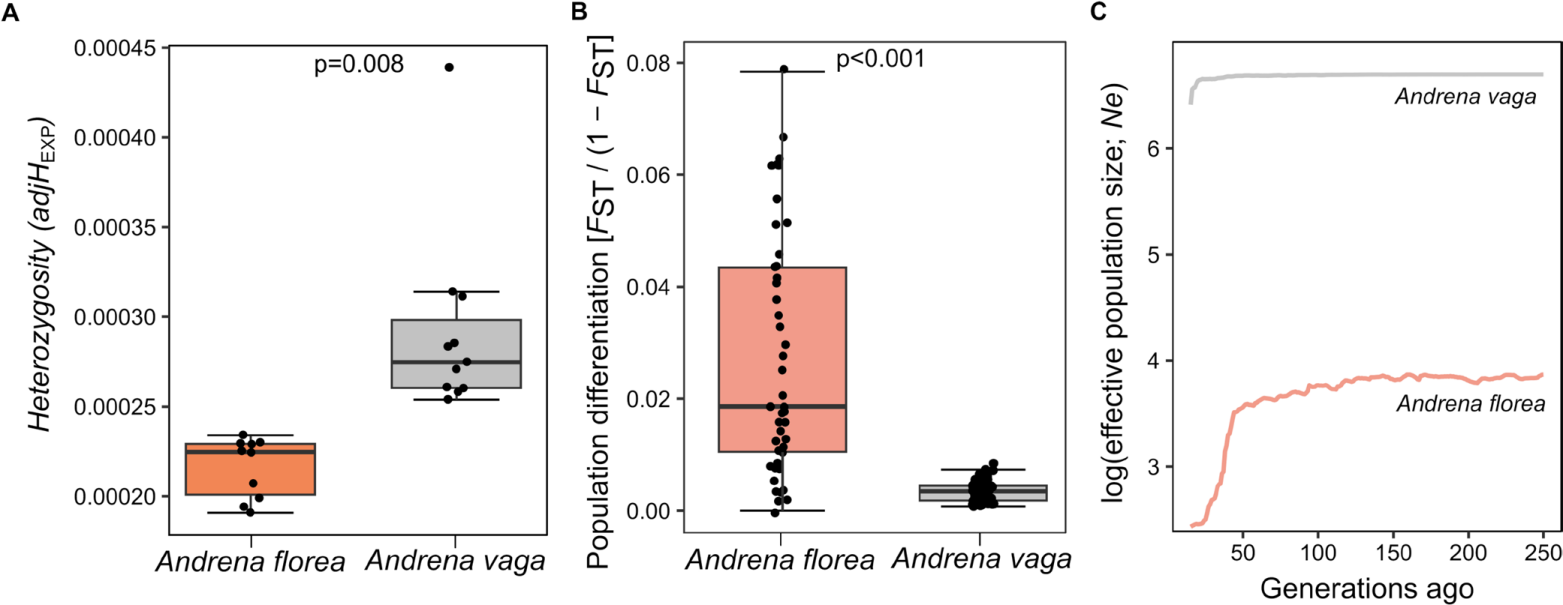
Box plots showing (A) genome‐wide genetic diversity, (B) population genetic differentiation and (C) recent demographic (*Ne*) trajectories across species. Boxes represent the interquartile range, with the horizontal line indicating the median. Whiskers extend to 1.5× the interquartile range.

Neutral genomic variation and demographic patterns differed between species. Genome‐wide genetic diversity (*adjH*_EXP_) ranged from 0.000191 to 0.000234 for *A. florea* and from 0.000254 to 0.000439 for *A. vaga* (Tables S1 and S2). Overall, *A. vaga* exhibited significantly higher genetic diversity than *A. florea* (LM; *t*‐value = 2.928, *p* = 0.008; Figure 2A).

**FIGURE 3:**
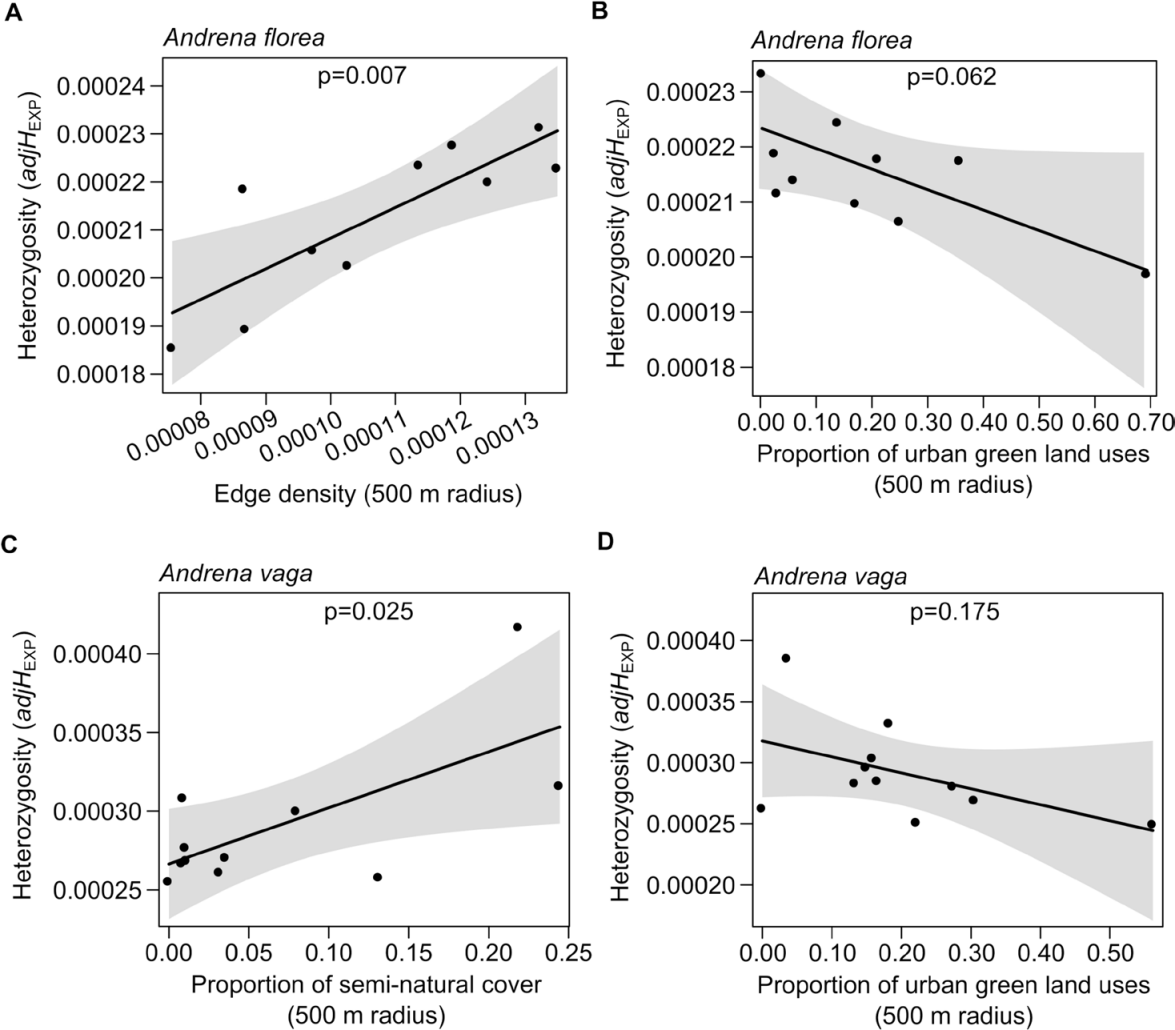
Relationships between *A. florea* heterozygosity and (A) edge density and (B) proportion of urban green land uses. Relationships between *A. vaga* heterozygosity and (C) proportion of semi‐natural cover and (D) proportion of urban green land uses. Black lines correspond to predicted relationships and shaded areas to 95% confidence intervals.

Impervious surface cover showed no significant effect on genetic diversity in either species ( *A. florea* : *t* = −0.194, *p* = 0.851; *A. vaga* : *t* = −0.426, *p* = 0.68; Figure S1). Landscape‐based models identified different predictors of genetic diversity between species. In *A. florea* , genetic diversity was positively associated with edge density (LM: *z* = 3.743, *p* = 0.007, *R* ^2^ = 0.54; Figure 3A; Table 1) and negatively, though not significantly so, with the proportion of urban green land uses (LM: *z* = 2.210, *p* = 0.062, *R* ^2^ = 0.15; Figure 3B; Table 1). In *A. vaga* , genetic diversity increased with the proportion of semi‐natural cover (LM: *z* = 2.299, *p* = 0.025, *R* ^2^ = 0.40; Figure 3C; Table 1) and decreased, though not significantly so, with the proportion of urban green land uses (LM: *z* = 1.355, *p* = 0.175, *R* ^2^ = 0.18; Figure 3D; Table 1).

**TABLE 1:** Model selection statistics and model‐averaged coefficients (conditional average) for *A. florea* and *A. vaga* genome‐wide genetic diversity (*adjH*_EXP_).

Response	Model	AIC	ΔAIC	Weight	Predictor	*z* value	*p*	*R* ^2^
*A. florea* *adjH*_EXP_	~Edge density (500 m radius) + proportion of urban green land uses (500 m radius)	−196.7	0.00	0.703	Edge density (500 m radius)	3.743	0.007	0.54
					Proportion of urban green land uses (500 m radius)	2.110	0.062	0.15
*A. vaga adjH*_EXP_	~Semi‐natural cover (500 m radius) + proportion of urban green land uses (500 m radius)	−188.09	0.00	0.34	Semi‐natural cover (500 m radius)	2.229	0.025	0.40
	~Semi‐natural cover (500 m radius)	−187.06	1.03	0.20	Proportion of urban green land uses (500 m radius)	1.355	0.175	0.18

**FIGURE 4:**
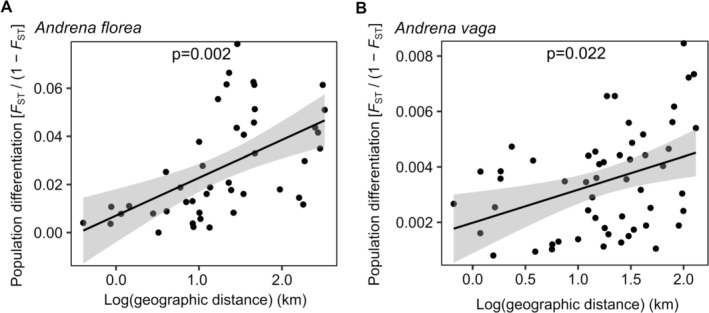
Relationship between pairwise genetic distance, measured as *F*_ST_/(1−*F*_ST_) and log‐geographic distance for (A) *A. florea* and (B) *A. vaga*. Black lines correspond to predicted relationships and shaded areas to 95% confidence intervals.

**FIGURE 5:**
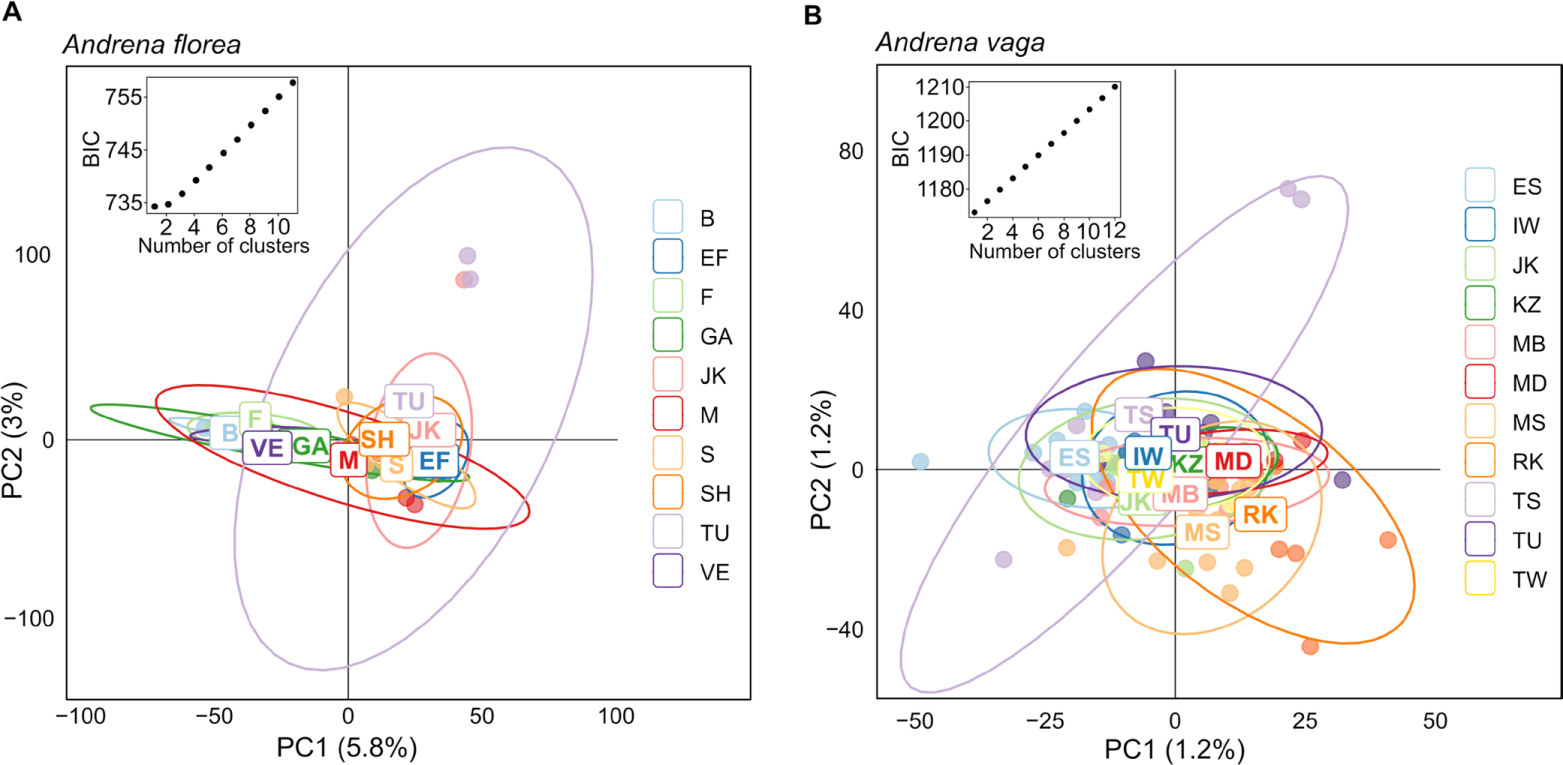
Number of population genetic clusters based on *find.clusters* and the BIC criterion and principal coordinate analysis (PCoA) scatter plot for (A) *A. florea* and (B) *A. vaga* .

Genetic differentiation between populations was low, but significantly greater than zero in both species ( *A. florea* : mean *F*_ST_ = 0.0258, 95% CI = 0.0252–0.0265; *A. vaga* : mean *F*_ST_ = 0.0034, 95% CI = 0.0031–0.0037; Tables S6 and S7). After accounting for geographic distances, *A. florea* had higher *F*_ST_ compared to *A. vaga* (Weighted least squares model; *t*‐value = 10.374, *p* < 0.001; Figure 2B). Isolation by distance was detected in both species (*A. florea: r* = 0.539, *p* = 0.002; *A. vaga* : *r* = 0.378, *p* = 0.022; Figure 4), although clustering analyses indicated no strong population structure (*K* = 1 for both species; Figure 5). This result was further supported by PCoA, which also showed no clear genetic structuring in either species (Figure 5).

Demographic reconstructions revealed contrasting recent histories. *Andrena florea* showed a marked decline in *Ne* beginning ~50 generations ago, consistent with a bottleneck, whereas *A. vaga* maintained relatively stable *Ne* through time. Contemporary *Ne* was substantially higher in *A. vaga* than in *A. florea* .

### 3.2 Genotype–Environment Associations and Haplotype‐Based Selection Scans (H2–H3)

**FIGURE 6:**
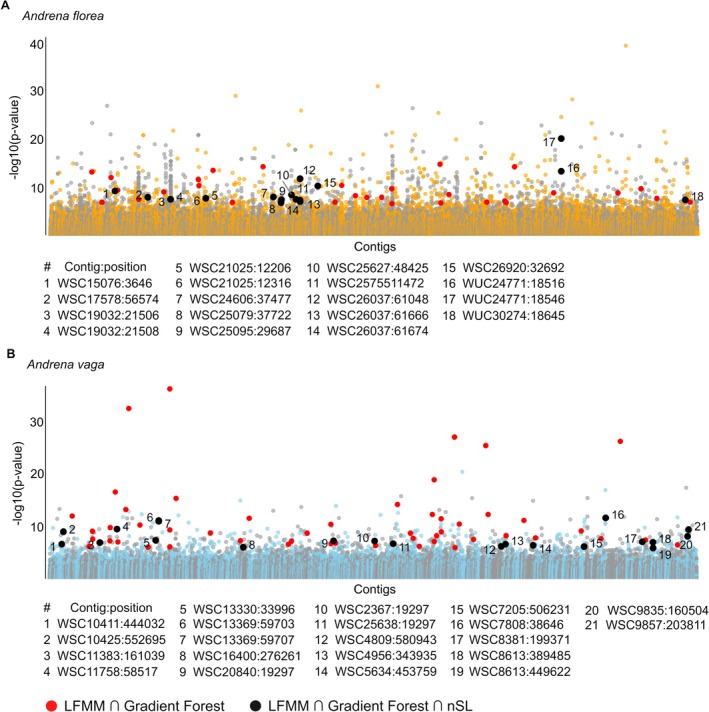
Genomic signals of genetic divergence associated with urbanisation in (A) *A. florea* and (B) *A. vaga* . −log_10_ *p*‐values from LFMM are presented in the y‐axis and contigs in the x‐axis of the Manhattan plots. Urbanisation‐associated loci (outliers) detected using both LFMM and Gradient Forest analyses (LFMM ∩ GF) are highlighted in red. Loci overlapping regions showing signatures consistent with selection (LFMM ∩ GF ∩ nSL) are highlighted in black and numbered. The numbers indicate the position of the contig to which they belong.

The LFMM analyses identified 1361 SNPs in *A. florea* (0.12% of the full SNP dataset, Table S8) and 550 SNPs in *A. vaga* (0.10% of the full SNP dataset, Table S9) associated with impervious surfaces. The GF analyses identified 559 SNPs in *A. florea* (0.05% of the full SNP dataset, Table S10) and 406 SNPs in *A. vaga* (0.07% of the full SNP dataset, Table S11). A total of 50 SNPs in *A. florea* and 74 SNPs in *A. vaga* were shared between methods (LFMM ∩ GF), exceeding expectations under random overlap (hypergeometric test, *p* < 0.001; Figure 6; Figures S2 and S3; Tables S12 and S13).

Haplotype‐based nSL analyses revealed signatures of positive selection in both species, identifying 1036 candidate sweep regions in *A. florea* and 1013 in *A. vaga* . In *A. florea* , 18 of the 50 SNPs identified by LFMM ∩ GF overlapped with sweep regions, while in *A. vaga* , 21 of 74 SNPs showed such overlap (Figure 6; Tables S12 and S13). In both species, overlaps were greater than expected by chance (permutation tests, *p* < 0.001).

**FIGURE 7:**
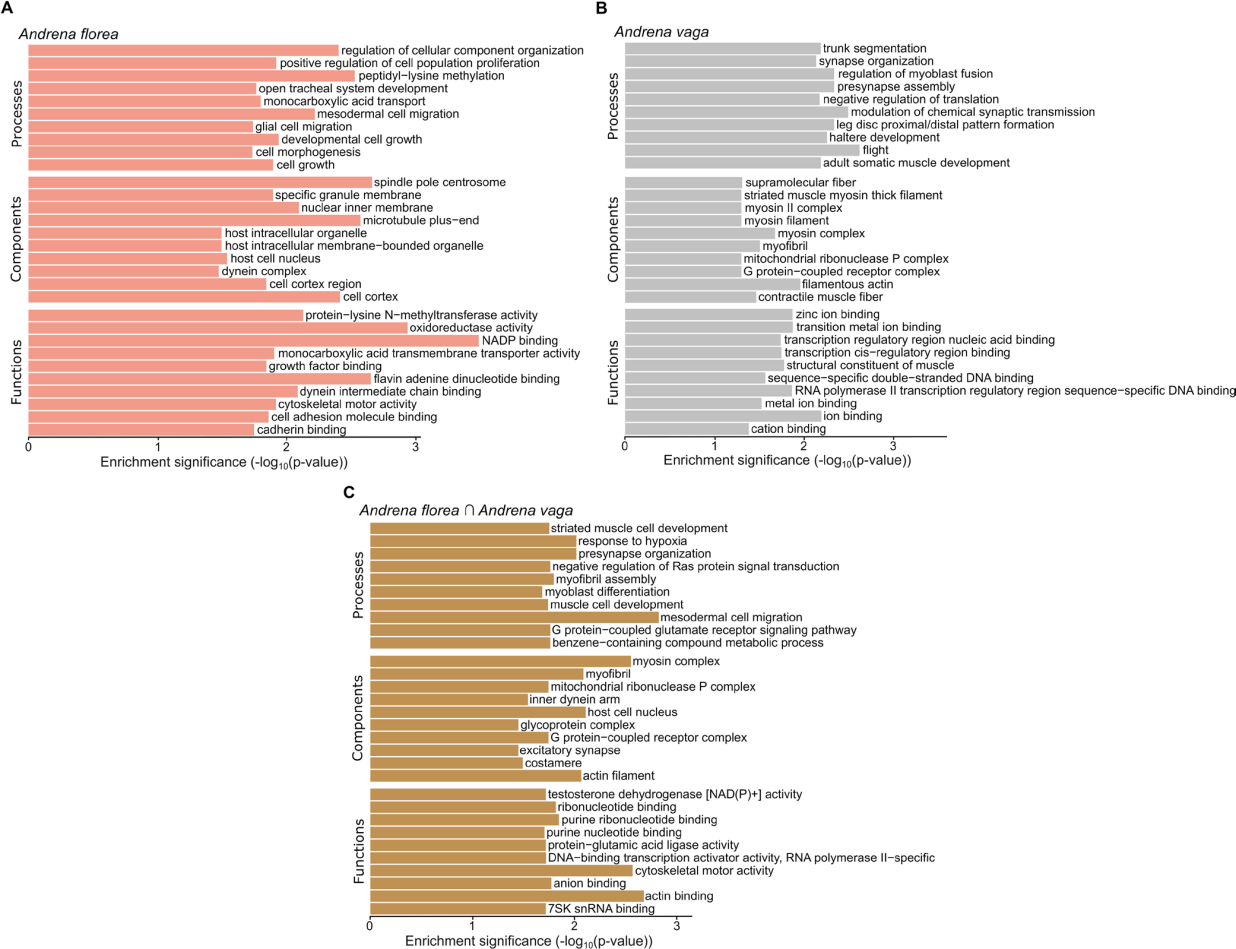
Bar plots show the top significantly enriched GO terms (*p* < 0.05) in (A) *Andrena florea* , (B) *Andrena vaga* and (C) shared between both species. GO terms are grouped by ontology category—Biological Process, Molecular Function, and Cellular Component—shown on the y‐axis. The x‐axis shows enrichment significance as –log_10_(*p*‐value) from a Fisher's exact test, where higher values indicate stronger over‐representation of a GO term among environmentally associated loci compared to the genomic background. Shared GO terms in panel C represent functional categories significantly enriched in both species, highlighting convergence in environmentally associated genomic responses at the level of biological pathways.

We identified 92 genes associated with urban intensity‐linked SNPs (LFMM ∩ GF) in *A. florea* and 121 in *A. vaga* (Tables S14 and S15). These genes are associated with biological processes, including responses to heat and oxidative stress, neural function and behaviour, metabolism, development and immune response (Tables S14 and S15). Gene ontology enrichment analyses supported these patterns. In *A. florea* , enriched terms were primarily related to cell growth, morphogenesis and cellular organisation, as well as nervous system and tracheal development. Molecular functions were dominated by oxidoreductase activity, cofactor binding and transport functions (Figure 7A; Table S16). In *A. vaga* , enriched categories were mainly associated with muscle development, structural components and neural signalling. Additional enrichment for ion‐binding and transcription‐related functions was also observed (Figure 7B; Table S17).

Functional patterns were similar for the subset of loci overlapping both GEA and selective sweep regions (LFMM ∩ GF ∩ nSL), supporting the robustness of these functional associations (Tables S18 and S19).

### 3.3 Cross‐Species Functional Convergence in Environmentally Associated Genomic Responses (H4)

*Andrena florea* and *A. vaga* shared 14 candidate orthologue sequences, exceeding expectations under random overlap (hypergeometric test, *p* < 0.001; Table S20). Across both species, 35 genes associated with urban intensity were identified, involved in processes such as muscle function, neurodevelopment, sensory systems, metabolism and stress responses (Table S21). Gene ontology enrichment analyses revealed overlap in functional pathways related to muscle development and contraction, cytoskeletal organisation, neural signalling and stress responses (Figure 7C; Table S22). Semantic similarity analyses further supported functional convergence. The combined similarity across GO categories was moderate (mean Wang similarity = 0.38) and significantly greater than expected by chance (permutation test, *p* = 0.01), indicating non‐random overlap in functional responses between species.

## 4 Discussion

In this study, we used a comparative population and landscape genomic framework to examine how genomic variation in urban landscapes is shaped by the interaction between demographic and selective processes in two solitary bee species that differ in key ecological traits. Our results show that genomic patterns in urban systems emerge from the interplay between landscape configuration, environmental gradients and species‐specific ecology—rather than urban intensity alone. Neutral genomic patterns differed markedly between species despite weak population structure in both, highlighting the role of species‐specific ecological traits in shaping connectivity, genetic drift and demographic patterns (H1). We further detected loci associated with urban intensity that overlapped with genomic regions showing signatures consistent with selection, demonstrating that environmentally structured genomic variation can arise even in the presence of gene flow (H2–H3). Finally, despite divergent demographic patterns, candidate genes and functional pathways showed partial convergence, suggesting that shared urban environmental gradients can be associated with partly similar functional genomic responses across species (H4).

### 4.1 Genetic Diversity, Divergence, Population Structure and Demographic Trends (H1)

Consistent with H1, genome‐wide genetic diversity was not strongly associated with impervious surface cover in either species, supporting the expectation that mobile bee species may experience cities as heterogeneous yet permeable environments. Instead, genetic diversity varied with landscape features related to habitat configuration and resource distribution, indicating that connectivity and habitat quality played a more prominent role than urban intensity per se.

Within this general pattern, the two species exhibited distinct, trait‐mediated associations between genetic diversity and landscape heterogeneity. In *A. florea* , genetic diversity increased with edge density, likely reflecting its strong dependence on *Bryonia* species, which occur in structurally complex transitional habitats such as hedgerows, roadside verges, riparian corridors (e.g., rivers and canals) and woodland edges (Banaszak et al. 2018; Brandes 1995; Westrich 2019). These habitats likely support both foraging and nesting, as *A. florea* has been reported to nest in proximity of *Bryonia* plants (Polidori and Federici 2019), potentially supporting larger local population sizes. Additionally, *A. florea* exhibits mating behaviour closely tied to its host plants, which may further concentrate reproductive activity in these edge habitats (Cilia et al. 2025; Polidori and Federici 2019). This spatial coupling of foraging, nesting and reproduction likely enhances connectivity among suitable sites (Vasiliev and Greenwood 2023).

In contrast, genetic diversity in *A. vaga* was positively associated with semi‐natural cover, highlighting the importance of ecologically rich habitats such as meadows and shrubby open areas for supporting genetically diverse populations. These habitats likely offer stable and abundant floral resources as well as suitable nesting opportunities, supporting larger and more stable populations while facilitating gene flow across the urban matrix (Banaszak‐Cibicka and Żmihorski 2011; Theodorou, Herbst, et al. 2020).

The lack of a strong relationship between genetic diversity and urban intensity in either species suggests that sealed surfaces may not act as strict barriers to movement for these species. Instead, this pattern likely reflects ongoing dispersal across urban green spaces, particularly for flying insects exploiting spatially distributed resources. This interpretation is consistent with previous studies showing that some bee species can persist in highly urbanised environments when suitable habitats are available (Johnson and Munshi‐South 2017; Schleimer et al. 2024; Soro et al. 2017; Theodorou et al. 2018).

A more nuanced pattern emerged when considering different types of urban green spaces. Managed green land uses (i.e., parks, cemeteries, allotment gardens and recreation grounds) showed a weak, non‐significant negative association with genetic diversity in both species, potentially reflecting variation in habitat quality. Intensively managed green spaces often provide limited resources for oligolectic, ground‐nesting bees (Baldock et al. 2019; Sobieraj‐Betlińska and Twerd 2024), although both species can occur in such environments. *Andrena vaga* is known to readily colonise such urban green land uses (Gardein et al. 2025). Less is known about the nesting habits of *A. florea* , but it appears to prefer compacted soils created by trampling and vehicle traffic, such as those found on sports fields (Westrich 2019). In such settings, long‐established nesting sites may promote philopatry, increasing relatedness among individuals and strengthening the effects of genetic drift. By contrast, semi‐natural patches and ecotones are more likely to be recolonised from multiple sources, promoting gene flow and helping to maintain higher genetic diversity. Although these associations are weak, they highlight the importance of distinguishing among types of urban green spaces when evaluating habitat quality. Moreover, they suggest that management interventions aimed at increasing the availability of host plants, nesting opportunities and habitat connectivity could support genetically diverse populations of solitary, diet‐specialised bees.

Across both species, genetic differentiation was low and clustering analyses revealed no strong population structure, consistent with ongoing gene flow and/or large effective population sizes, as reported for other bee species in urban environments (Schleimer and Frantz 2025). However, low but statistically significant *F*_ST_ values indicate subtle population subdivision. This may partly reflect residual kin structure, given that solitary, ground‐nesting females often exhibit natal philopatry and limited dispersal (Bischoff et al. 2003; Friedel et al. 2017). Additionally, spatial variation in local effective population sizes, driven by heterogeneity in floral and nesting resources, can influence *F*_ST_ independently of gene flow (Prunier et al. 2017). These effects were more pronounced in *A. florea* , which exhibited higher *F*_ST_ and stronger isolation by distance, consistent with more restricted dispersal or smaller effective population size compared to *A. vaga* . In contrast, the larger body size and reliance on more widely distributed host plants of *A. vaga* likely contributed to greater landscape permeability. Indeed, previous studies in Central Europe found little or no genetic structuring in *A. vaga* , even across natural landscapes with populations separated by over 300 km (Černá et al. 2013; Exeler et al. 2008). The higher genome‐wide genetic diversity observed in *A. vaga* is consistent with this interpretation, although differences in sampling strategy ( *A. florea* sampled on flowers versus *A. vaga* sampled at nesting aggregations) should be considered when comparing species. Sampling on flowers likely captures foraging individuals from multiple nesting sites, whereas sampling at aggregations may over‐represent locally related individuals. Such differences can influence estimates of genetic diversity and relatedness, and may contribute to the observed contrasts between species. Nevertheless, our results align with previous research showing that urbanisation does not uniformly reduce gene flow or genetic diversity in bees (Ballare and Jha 2020; Schleimer et al. 2024; Soro et al. 2017; Theodorou et al. 2018). Our results also reinforce the idea that species‐specific ecological traits such as body size, nesting behaviour and diet specialisation are central to shaping genetic responses to urban environments (López‐Uribe et al. 2019; Miles et al. 2019).

Finally, demographic reconstructions reinforce these species‐specific neutral genomic patterns. *Andrena florea* exhibited a pronounced decline in *Ne* starting approximately 50 generations ago, whereas *A. vaga* showed relatively stable *Ne* throughout. The decline in *A. florea* coincides with periods of intensified land‐use change and landscape fragmentation in Germany (Niedertscheider et al. 2014), which may have reduced its habitat availability and connectivity. In contrast, the stability of *A. vaga* may reflect its broader ecological tolerance and reliance on more widespread and persistent host plants (*Salix* spp.), which provide relatively stable resources over time (Kuzovkina and Volk 2009). *Salix* spp. are long‐lived and thus provide a reliable food resource for *A. vaga* aggregations over decades, whereas *Bryonia* are comparatively short‐lived herbaceous plants frequently eradicated due to their toxicity, potentially contributing to greater temporal instability in host‐plant availability for *A. florea* .

### 4.2 Genotype–Environment Associations and Haplotype‐Based Selection Scans (H2–H3)

Supporting our prediction under H2, that spatially structured urban environmental gradients can be associated with detectable genomic variation even in partially permeable systems (Charmantier et al. 2024; Johnson and Munshi‐South 2017), both species exhibited genomic variation associated with urban intensity despite weak population structure and ongoing gene flow. Genotype–environment association analyses identified numerous SNPs associated with impervious surface cover, with a subset consistently detected across complementary methods (LFMM ∩ GF). These patterns are consistent with selective pressures such as thermal variability, pollution and altered resource distribution acting across urban gradients, while gene flow simultaneously homogenises neutral variation. Under such conditions, genotype–environment associations reflect spatially structured environmental filtering rather than complete population divergence, a pattern increasingly reported in urban genomic studies of mobile taxa (Babik et al. 2023; Bourgeois et al. 2025; Salmón et al. 2021; Theodorou et al. 2018).

In line with H3, haplotype‐based scans further identified numerous genomic regions exhibiting signatures consistent with positive selection. This non‐random overlap between urban intensity‐associated loci and nSL outlier regions indicates that some of these genomic regions may be shaped by non‐neutral processes consistent with spatially structured selection acting on standing genetic variation. Given the weak population structure observed in both species, these patterns are consistent with localised allele frequency shifts along environmental gradients rather than classic hard selective sweeps driven by strong demographic isolation.

Functional annotation provided further insight into the biological processes potentially associated with these patterns. In *A. florea* , several urban intensity‐associated candidate genes were functionally related to metabolic regulation, neural processes and stress response pathways. For example, genes involved in mitochondrial function, detoxification and oxidative stress tolerance (e.g., ATP synthase subunits (Xu et al. 2015), cytochrome P450‐related pathways and multidrug resistance‐associated proteins (Bhat et al. 2024; Fent et al. 2020; Iyanagi 2005)) may reflect physiological responses to urban stressors such as pollutants, thermal variability and altered resource availability. Additional candidates linked to neural development and behavioural regulation, including genes associated with synaptic function and neuropeptide signalling (e.g., CCHamide‐1 (Shahid et al. 2024)), could be relevant in the context of foraging in spatially heterogeneous environments where resource distribution is patchy and temporally variable. Structural and cytoskeletal genes were also identified (e.g., dynactin subunit 1 (Bercier et al. 2019), and WASH complex subunit 4 (Fokin and Gautreau 2021)), which may be associated with sustained flight performance and locomotor demands in fragmented urban habitats.

In *A. vaga* , candidate loci associated with urban intensity were similarly linked to pathways involved in muscle function, neurodevelopment and metabolic stress responses. Several genes related to locomotion and muscle structure were identified (e.g., myosin heavy chain (Wells et al. 1996), myophilin and muscle‐specific protein 20 (Ayme‐Southgate et al. 1989)), which may be relevant for species navigating patchy urban landscapes where foraging distances and resource distribution can vary spatially. Neural‐related candidates, including genes involved in synaptic plasticity and sensory processing (e.g., neuroligin 4 (Bang and Owczarek 2013), metabotropic glutamate receptor (Kucharski et al. 2007) and Krueppel‐like factor 6 (Laitman et al. 2016)), may reflect the cognitive demands of exploiting complex and heterogeneous urban landscapes. In addition, genes associated with oxidative stress regulation and detoxification pathways (e.g., superoxide dismutase 1 (Tsang et al. 2014) and cytochrome P450 304a1 (Shelomi 2022)) were detected, consistent with potential exposure to pollutants and elevated thermal stress in urban settings. However, the functional interpretation of candidate loci remains tentative, and these associations should be viewed as hypotheses about underlying biological processes rather than direct evidence of adaptation.

### 4.3 Cross‐Species Functional Convergence in Environmentally Associated Genomic Responses (H4)

Consistent with H4, our results provide evidence for partial convergence in environmentally associated genomic responses to urban intensity between species. Despite clear differences in neutral genomic patterns and demographic history, *A. florea* and *A. vaga* shared a subset of orthologous genes associated with urban intensity, suggesting that exposure to similar urban environmental gradients may be associated with overlapping genomic responses, regardless of species‐specific differences in connectivity and genetic drift.

Shared candidate genes were broadly related to functional categories such as muscle performance, neural function and metabolic regulation. This pattern was supported by semantic similarity analyses of enriched Gene Ontology terms, which revealed significantly greater functional overlap between species than expected by chance. Together, these results indicate that urban intensity–associated genomic variation in both species converges on similar functional pathways, even though the specific genes involved differ. This pattern does not imply identical adaptive trajectories, but rather suggests that similar environmental pressures in urban landscapes may be associated with changes in genomic regions linked to locomotion, behavioural flexibility and physiological stress responses. These functional dimensions are likely important for mobile insects navigating spatially structured resources and variable environmental conditions.

However, these findings should be interpreted cautiously. Shared candidate genes and functional similarity do not constitute direct evidence of adaptive convergence, and our analyses are based on a single urban landscape. As such, we cannot determine whether similar genomic responses would arise across independent urban systems. Future comparative studies across replicated landscapes will be essential to assess the generality of these patterns.

### 4.4 Conclusions

Our results show that genomic patterns in urban systems cannot be explained by urban intensity alone, but instead emerge from the interplay between landscape configuration, environmental gradients and species‐specific ecology. In particular, species‐specific ecological traits shaped neutral genomic patterns, with differences in genetic diversity, population differentiation and demographic history reflecting variation in connectivity and effective population size rather than direct effects of urbanisation. At the same time, both species exhibited environmentally associated genomic variation linked to urban environmental gradients, with signals consistent with selection despite ongoing gene flow. Taken together, these findings demonstrate that species exposed to the same urban landscape can follow divergent demographic trajectories while exhibiting partly convergent genomic responses. This highlights that ecological traits mediate how populations experience and respond to urban environments, shaping both the strength of genetic drift and the genomic signature of environmental gradients. More broadly, predicting which species can maintain viable populations in urban landscapes will require moving beyond measures of urban intensity toward a more mechanistic understanding of how species‐specific ecology, landscape configuration and evolutionary processes interact across heterogeneous urban systems.

## Author Contributions

Lucie M. Baltz participated in study design, collected and analysed the data, and drafted the manuscript. Hanna Gardein and Henri Greil participated in bee sampling. Hanna Gardein participated in land use and aggregation size data collection. Robert J. Paxton participated in study design and data interpretation, and Panagiotis Theodorou participated in study design, drafted the manuscript, analysed the data and data interpretation. All authors contributed in drafting and finalising the manuscript.

## Funding

This study is part of the BeesUp project, which is funded by the German Federal Agency for Nature Conservation (within the Federal Biological Diversity Programme) with funds from the Federal Ministry for the Environment, Nature Conservation, Nuclear Safety and Consumer Protection (Grant Numbers: 3520685A29 and 3520685C29).

## Disclosure

Benefit sharing statement: Benefits generated: Benefits from this research accrue from the sharing of our data and results on public databases, as described above.

## Conflicts of Interest

The authors declare no conflicts of interest.

## Supporting information


**Figure S1:** (A) Relationship between *A. florea* heterozygosity and proportion of impervious surfaces. (B) Relationship between *A. vaga* heterozygosity and proportion of impervious surfaces. Black lines correspond to predicted relationships and shaded areas to 95% confidence intervals. ns, not significant, *p* > 0.05.
**Figure S2:** Venn diagram of the number of outlier loci identified by each of the two methods applied (i.e., LFMM and Gradient forest) for *Andrena florea* .
**Figure S3:** Venn diagram of the number of outlier loci identified by each of the two methods applied (i.e., LFMM and Gradient forest) for *Andrena vaga* .
**Table S1:** mec70370‐sup‐0001‐Supinfo.docx. *Andrena florea* population ID, sample size, coordinates, autosomal expected heterozygosity (*adjH*_EXP_ = He * number of polymorphic loci/number of all callable sites), edge density and proportion of the main land cover classes.
**Table S2:** mec70370‐sup‐0001‐Supinfo.docx. *Andrena vaga* population ID, sample size, coordinates, autosomal expected heterozygosity (*adjH*_EXP_ = He * number of polymorphic loci/number of all callable sites), edge density, proportion of the main land cover classes, water body edges and the estimated number of nests.
**Table S3:** Summary of genome assembly quality and statistics for *A. florea* and *A. vaga* , including completeness assessment results, length statistics and composition.
**Table S4:** mec70370‐sup‐0001‐Supinfo.docx. *Andrena florea* sequencing depth, genome coverage and number of read pairs per individual.
**Table S5:** mec70370‐sup‐0001‐Supinfo.docx. *Andrena vaga* sequencing depth, genome coverage and number of read pairs per individual.
**Table S6:** mec70370‐sup‐0001‐Supinfo.docx. *Andrena florea* estimated pairwise *F*_ST_ between all population pairs (above diagonal). *p*‐values from 1000 bootstraps (below diagonal).
**Table S7:** mec70370‐sup‐0001‐Supinfo.docx. *Andrena vaga* estimated pairwise *F*_ST_ between all location pairs (above diagonal). *p*‐values from 1000 bootstraps (below diagonal).
**Methods S1** Draft reference genome assemblies for *A. florea* and *A. vaga*.

**Table S8:** Urban intensity‐associated SNPs detected using LFMM in *Andrena florea* . The contig and SNP position are reported.
**Table S9:** Urban intensity‐associated SNPs detected using LFMM in *Andrena vaga* . The contig and SNP position are reported.
**Table S10:** Urban intensity‐associated SNPs detected using Gradient forest in *Andrena florea* . The contig and SNP position are reported.
**Table S11:** Urban intensity‐associated SNPs detected using Gradient forest in *Andrena vaga* . The contig and SNP position are reported.
**Table S12:** Urban intensity‐associated SNPs detected using LFMM and Gradient forest (LFMM ∩ GF) in *Andrena florea* . The contig and SNP position are reported.
**Table S13:** Urban intensity‐associated SNPs detected using LFMM and Gradient forest (LFMM ∩ GF) in *Andrena vaga* . The contig and SNP position are reported.
**Table S14:** Annotation of the genes identified to be associated with urban intensity using LFMM ∩ GF in *Andrena florea* . The contig, SNP position, gene and protein are reported.
**Table S15:** Annotation of the genes identified to be associated with urban intensity using LFMM ∩ GF in *Andrena florea* . The contig, SNP position, gene and protein are reported.
**Table S16:** Gene ontology enrichment analysis of LFMM ∩ GF urban intensity‐associated SNPs in *Andrena florea* . GO.ID: GO.ID: Identifier for the Gene Ontology term. Term: Description or name of the GO term associated with the GO.ID. Annotated: The total number of genes in the background set that are annotated with the specific GO term. Significant: Number of genes in our list of interest that are annotated with the specific GO term. Expected: This is the number of genes one would expect to be annotated with the specific GO term in our list of interest if there were no enrichment. It is calculated based on the proportion of genes annotated with the GO term in the background set. *p*‐value: Statistical significance of the enrichment of the GO term in our list of interest.
**Table S17:** Gene ontology enrichment analysis of LFMM ∩ GF urban intensity‐associated SNPs in *Andrena vaga* . GO.ID: Identifier for the Gene Ontology term. Term: Description or name of the GO term associated with the GO.ID. Annotated: The total number of genes in the background set that are annotated with the specific GO term. Significant: Number of genes in our list of interest that are annotated with the specific GO term. Expected: This is the number of genes one would expect to be annotated with the specific GO term in our list of interest if there were no enrichment. It is calculated based on the proportion of genes annotated with the GO term in the background set. *p*‐value: Statistical significance of the enrichment of the GO term in our list of interest.
**Table S18:** Gene ontology enrichment analysis of LFMM ∩ GF ∩ nSL SNPs in *Andrena florea* . GO.ID: GO.ID: Identifier for the Gene Ontology term. Term: Description or name of the GO term associated with the GO.ID. Annotated: The total number of genes in the background set that are annotated with the specific GO term. Significant: Number of genes in our list of interest that are annotated with the specific GO term. Expected: This is the number of genes one would expect to be annotated with the specific GO term in our list of interest if there were no enrichment. It is calculated based on the proportion of genes annotated with the GO term in the background set. *p*‐value: Statistical significance of the enrichment of the GO term in our list of interest.
**Table S19:** Gene ontology enrichment analysis of LFMM ∩ GF ∩ nSL SNPs in *Andrena vaga* . GO.ID: Identifier for the Gene Ontology term. Term: Description or name of the GO term associated with the GO.ID. Annotated: The total number of genes in the background set that are annotated with the specific GO term. Significant: Number of genes in our list of interest that are annotated with the specific GO term. Expected: This is the number of genes one would expect to be annotated with the specific GO term in our list of interest if there were no enrichment. It is calculated based on the proportion of genes annotated with the GO term in the background set. *p*‐value: Statistical significance of the enrichment of the GO term in our list of interest.
**Table S20:** Urban intensity‐associated sequence orthologues between *Andrena florea* and *Andrena vaga* . The species, contig ID, SNP position and corresponding orthologue group are reported.
**Table S21:** Annotation of the genes identified to be associated with urban intensity in both *Andrena florea* and *Andrena vaga* . The gene and protein are reported.
**Table S22:** Gene ontology enrichment analysis of LFMM ∩ GF urban intensity‐associated SNPs in *Andrena florea* and *Andrena vaga* . GO.ID: Identifier for the Gene Ontology term. Term: Description or name of the GO term associated with the GO.ID. Annotated: The total number of genes in the background set that are annotated with the specific GO term. Significant: Number of genes in our list of interest that are annotated with the specific GO term. Expected: This is the number of genes one would expect to be annotated with the specific GO term in our list of interest if there were no enrichment. It is calculated based on the proportion of genes annotated with the GO term in the background set. *p*‐value: Statistical significance of the enrichment of the GO term in our list of interest.

## Data Availability

Raw sequence data are available on the NCBI short read archives under the Bioproject IDs PRJNA1269256 and PRJNA1264677. SNP data (VCF format) are available on figshare (https://figshare.com/s/87a691a1f8e6449a6205) and command line scripts on github (https://github.com/panastheod/Andrena_molecular_ecology_genomics).
