# Supplementary material for: Different Paths, Similar Pressures: Divergent Drivers of Genetic Diversity Despite Convergent Genomic Signatures of Selection in Response to Urban Intensity in Two Oligolectic Bee Species
Source: Mol Ecol. 2026 May 8;35:e70370. doi: 10.1111/mec.70370 (PMC13156532; doi:10.1111/mec.70370)
Supplement: Supplementary file 1 — Figure S1: (A) Relationship between A. florea heterozygosity and proportion of impervious surfaces. (B) Relationship between A. vaga heterozygosity and proportion of impervious surfaces. Black lines correspond to predicted relationships and shaded areas to 95% confidence intervals. ns, not significant, p > 0.05. Figure S2: Venn diagram of the number of outlier loci identified by each of the two methods applied (i.e., LFMM and Gradient forest) for Andrena florea . Figure S3: Venn diagram of the number of outlier loci identified by each of the two methods applied (i.e., LFMM and Gradient forest) for Andrena vaga . Table S1: mec70370‐sup‐0001‐Supinfo.docx. Andrena florea population ID, sample size, coordinates, autosomal expected heterozygosity (adjH EXP = He * number of polymorphic loci/number of all callable sites), edge density and proportion of the main land cover classes. Table S2: mec70370‐sup‐0001‐Supinfo.docx. Andrena vaga population ID, sample size, coordinates, autosomal expected heterozygosity (adjH EXP = He * number of polymorphic loci/number of all callable sites), edge density, proportion of the main land cover classes, water body edges and the estimated number of nests. Table S3: Summary of genome assembly quality and statistics for A. florea and A. vaga , including completeness assessment results, length statistics and composition. Table S4: mec70370‐sup‐0001‐Supinfo.docx. Andrena florea sequencing depth, genome coverage and number of read pairs per individual. Table S5: mec70370‐sup‐0001‐Supinfo.docx. Andrena vaga sequencing depth, genome coverage and number of read pairs per individual. Table S6: mec70370‐sup‐0001‐Supinfo.docx. Andrena florea estimated pairwise F ST between all population pairs (above diagonal). p‐values from 1000 bootstraps (below diagonal). Table S7: mec70370‐sup‐0001‐Supinfo.docx. Andrena vaga estimated pairwise F ST between all location pairs (above diagonal). p‐values from 1000 bootstraps (below diagonal). Methods S1 Draft refere [file MEC-35-e70370-s002.docx]

Supplemental Information for:

**Different paths, similar pressures: divergent drivers of genetic diversity despite convergent genomic signatures of selection in response to urban intensity in two oligolectic bee species**

Lucie M. Baltz, Hanna Gardein, Henri Greil, Robert J. Paxton, Panagiotis Theodorou

**Contents**

[**Methods S1.** Draft reference genome assemblies for *A. florea* and *A. vaga* 2](#_Toc222576423)

[**Figure S1.** (A) Relationship between *A. florea* heterozygosity and proportion of impervious surfaces. (B) Relationship between *A. vaga* heterozygosity and proportion of impervious surfaces. Black lines correspond to predicted relationships and shaded areas to 95% confidence intervals. ns, not significant, p > 0.05. 4](#_Toc222576424)

[**Figure S2.** Venn diagram of the number of outlier loci identified by each of the two methods applied (i.e. LFMM and Gradient Forest) for *Andrena florea.* 5](#_Toc222576425)

[**Figure S3.** Venn diagram of the number of outlier loci identified by each of the two methods applied (i.e. LFMM and Gradient Forest) for *Andrena vaga.* 6](#_Toc222576426)

[**Table S1.** *Andrena florea* population ID, sample size, coordinates, autosomal expected heterozygosity (*adjH*_EXP_ = He * number of polymorphic loci / number of all callable sites), edge density and proportion of the main land cover classes. 7](#_Toc222576427)

[**Table S2.** *Andrena vaga* population ID, sample size, coordinates, autosomal expected heterozygosity (*adjH*_EXP_ = He * number of polymorphic loci / number of all callable sites), edge density, proportion of the main land cover classes, water body edges and the estimated number of nests. 8](#_Toc222576428)

[**Table S3.** Summary of genome assembly quality and statistics for *A. florea* and *A. vaga*, including completeness assessment results, length statistics and composition. 9](#_Toc222576429)

[**Table S4.** *Andrena florea* sequencing depth, genome coverage and number of read pairs per individual. 10](#_Toc222576430)

[**Table S5.** *Andrena vaga* sequencing depth, genome coverage and number of read pairs per individual. 12](#_Toc222576431)

[**Table S6**. *Andrena florea* estimated pairwise *F*_ST_ between all population pairs (above diagonal). P-values from 1000 bootstraps (below diagonal). 15](#_Toc222576432)

[**Table S7.** *Andrena vaga* estimated pairwise *F*_ST_ between all location pairs (above diagonal). P-values from 1000 bootstraps (below diagonal). 16](#_Toc222576433)

[**List of supplementary data tables in an Excel file** 17](#_Toc222576434)

[**References** 20](#_Toc222576435)

# **Methods S1.** Draft reference genome assemblies for *A. florea* and *A. vaga*

To generate draft reference genomes for *A. florea* and *A. vaga*, a single male of each species was collected using a hand net and prepared for whole-genome sequencing. DNA was extracted from the thorax using a digestion buffer (2% cetyltrimethylammonium bromide [CTAB], 20 mM disodium EDTA [Na₂EDTA 2H₂O], 1.4 M NaCl, 100 mM Tris-HCl, pH 8.0) and proteinase K (20 mg/mL), followed by extraction with a chloroform:isoamyl alcohol mixture (24:1). DNA concentration and integrity were assessed with a Qubit 3.0 fluorometer (Thermo Fisher Scientific, Waltham, MA, USA) and an Epoch spectrophotometer (BioTek, Winooski, USA). To generate reference genomes, we used both short paired-end Illumina reads and PacBio long-reads. Sequencing was conducted by Bioarker Technologies (BMK) GmbH (Münster, Germany), using the Pacific Biosciences Revio platform for HiFi reads and the NovaSeq X Plus system for Illumina sequencing. This process yielded 160,519,947 paired-end Illumina reads and 263,526 HiFi reads for *A. florea* and 205,580,425 paired-end Illumina reads and 814,461 HiFi reads for *A. vaga*.

Preprocessing of Illumina data involved adapter removal and quality filtering, discarding reads with a Phred score below 30. This was performed using BBDuk (BBMap v. 38.91, sourceforge.net/projects/bbmap/) with parameters set to tpe, tbo, qtrim = rl, trimq = 30. Hybrid genome assembly was conducted using two different assemblers: Spades v. 4.1.0 (Prjibelski et al., 2020) and WENGAN v. 0.2 (Minia3 short-read assembler in "M" mode) (Di Genova et al., 2021), applying default settings. The best genome assembly for each species was selected based on genome completeness, evaluated using BUSCO v. 5.8.3 (Seppey et al., 2019) with the Hymenoptera orthologue dataset and by evaluating length and composition statistics using gVolante (Nishimura et al., 2017). The optimal assembly for *A. florea*, produced by WENGAN, suggested a genome size of approximately 252 Mbp (GCA_052575755.1; Table S3), while the best assembly for *A. vaga*, also produced by WENGAN, estimated a genome size of approximately 288 Mbp (GCA_052575775.1; Table S3). The assemblies were cleaned using the NCBI foreign contamination screen (FCS) tool (Astashyn et al., 2024) and polished using NextPolish (Hu et al., 2020). Both assemblies demonstrated high completeness, recovering 91.17% (*A. florea*) and 95.24% (*A. vaga*) of conserved Hymenopteran orthologues, as assessed using BUSCO v. 5.8.3 (Table S3).


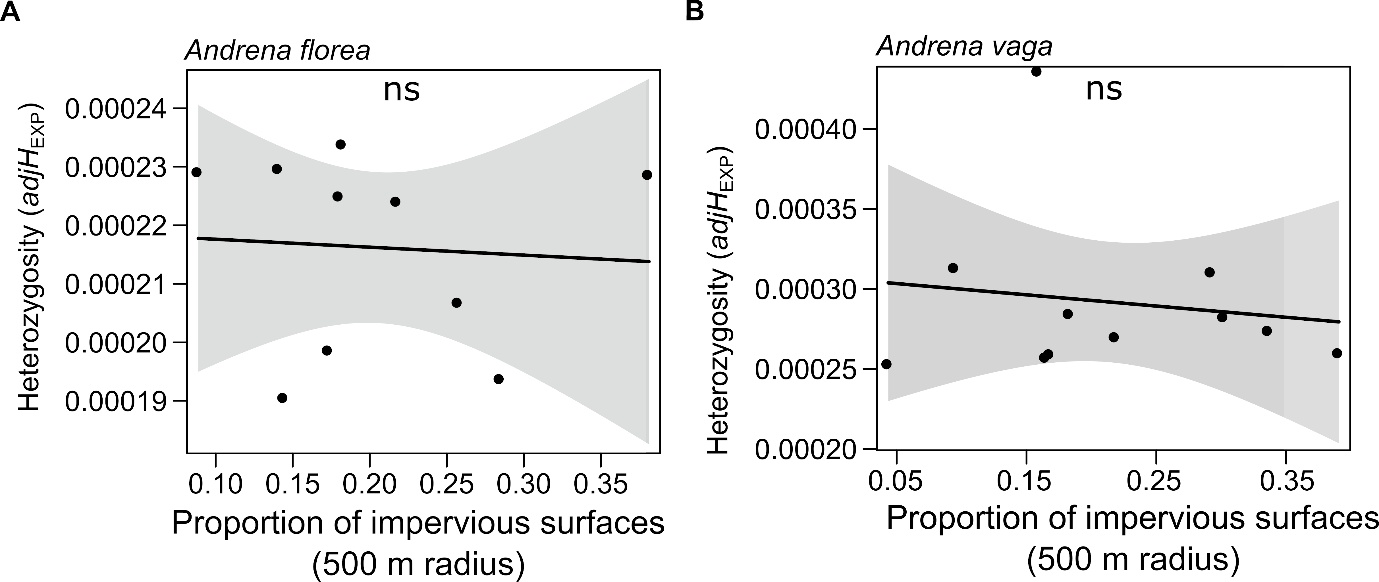


# **Figure S1.** (A) Relationship between *A. florea* heterozygosity and proportion of impervious surfaces. (B) Relationship between *A. vaga* heterozygosity and proportion of impervious surfaces. Black lines correspond to predicted relationships and shaded areas to 95% confidence intervals. ns, not significant, p > 0.05.


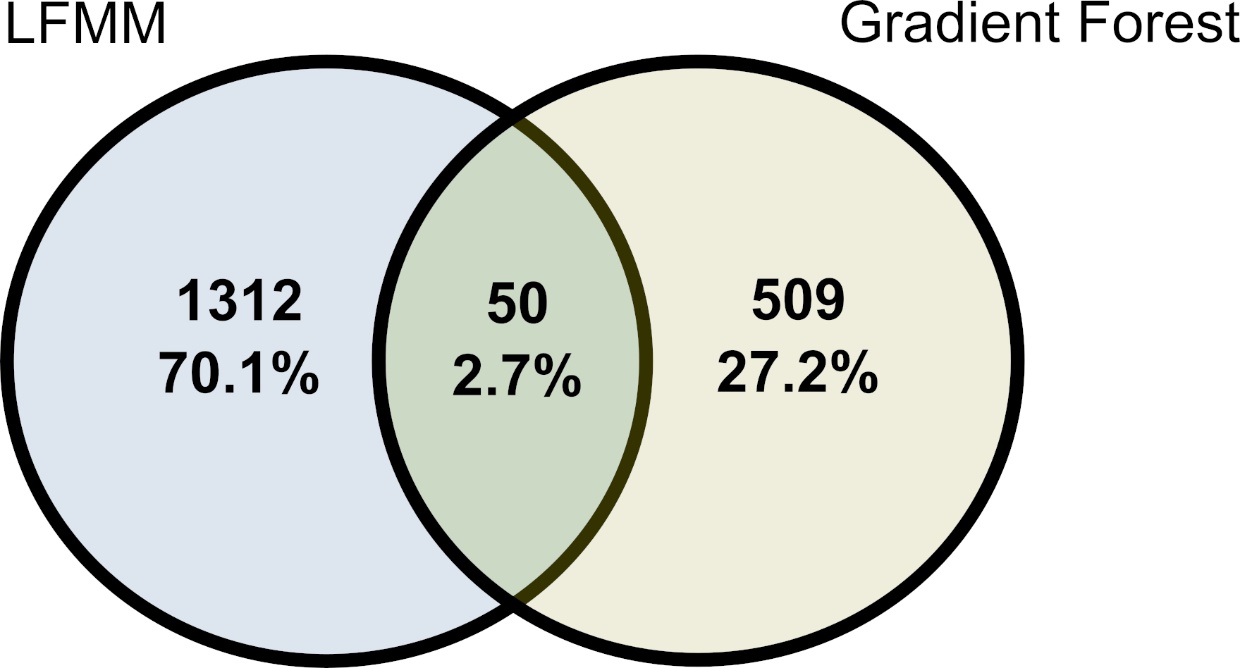


# **Figure S2.** Venn diagram of the number of outlier loci identified by each of the two methods applied (i.e. LFMM and Gradient Forest) for *Andrena florea.*


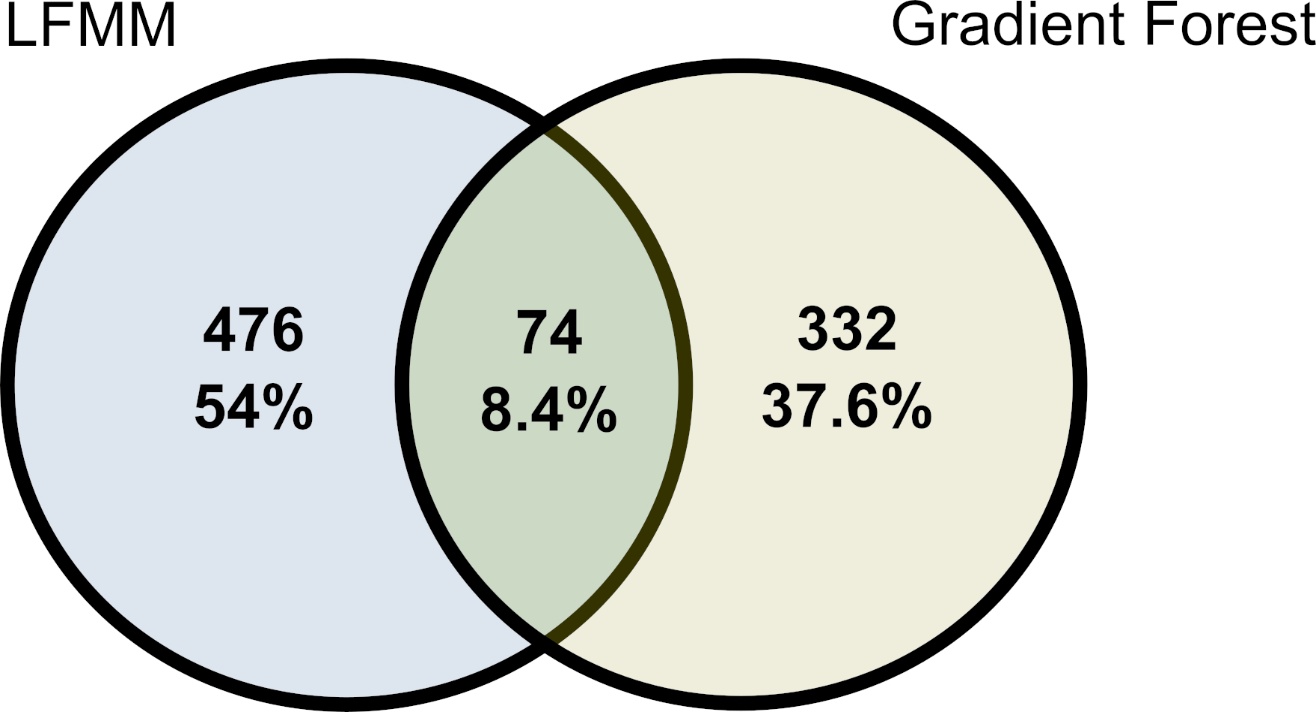


# **Figure S3.** Venn diagram of the number of outlier loci identified by each of the two methods applied (i.e. LFMM and Gradient Forest) for *Andrena vaga.*

# **Table S1.** *Andrena florea* population ID, sample size, coordinates, autosomal expected heterozygosity (*adjH*_EXP_ = He * number of polymorphic loci / number of all callable sites), edge density and proportion of the main land cover classes.

| Population ID | N | Latitude | Longitude | Expected heterozygosity (*adjH*_EXP_) | Edge density at 500 m radius | Proportion of impervious surfaces at 500 m radius | Proportion of green urban land uses at 500 m radius | Proportion of seminatural cover at 500 m radius |
| --- | --- | --- | --- | --- | --- | --- | --- | --- |
| B | 7 | 52.25107 | 10.52295 | 0.000191 | 0.0000973 | 0.144417 | 0.693677 | 0.004327 |
| EF | 7 | 52.28257 | 10.55977 | 0.000194 | 0.0000756 | 0.284855 | 0.060064 | 0.107625 |
| F | 7 | 52.2642 | 10.48912 | 0.000225 | 0.0001189 | 0.180289 | 0.357657 | 0.101658 |
| GA | 7 | 52.27199 | 10.52378 | 0.000229 | 0.0001137 | 0.381233 | 0.139415 | 0.007622 |
| JK | 7 | 52.27483 | 10.56514 | 0.000199 | 0.0000869 | 0.173312 | 0.030072 | 0.305942 |
| M | 7 | 52.29817 | 10.5087 | 0.000224 | 0.0001349 | 0.217787 | 0.250118 | 0.203367 |
| S | 7 | 52.27454 | 10.55529 | 0.000234 | 0.0001323 | 0.182273 | 0.210736 | 0.298122 |
| SH | 7 | 52.29657 | 10.54545 | 0.00023 | 0.0001243 | 0.140859 | 0.025812 | 0.293763 |
| TU | 7 | 52.28256 | 10.54422 | 0.000207 | 0.0001027 | 0.257577 | 0.171467 | 0.031968 |
| VE | 7 | 52.26966 | 10.38391 | 0.000229 | 0.0000866 | 0.088688 | 0.003046 | 0.281487 |

# **Table S2.** *Andrena vaga* population ID, sample size, coordinates, autosomal expected heterozygosity (*adjH*_EXP_ = He * number of polymorphic loci / number of all callable sites), edge density, proportion of the main land cover classes, water body edges and the estimated number of nests.

| Population ID | N | Latitude | Longitude | Expected heterozygosity (*adjH*_EXP_) | Edge density at 500 m radius | Proportion of impervious surfaces at 500 m radius | Proportion of green urban land uses at 500 m radius | Proportion of seminatural cover at 500 m radius | Amount of water body edges at 500 m radius | Estimated number of nests |
| --- | --- | --- | --- | --- | --- | --- | --- | --- | --- | --- |
| ES | 12 | 52.29006 | 10.55439 | 0.000439 | 0.0000850 | 0.159042 | 0.036065 | 0.218882 | 5566 | 33071.7 |
| IW | 11 | 52.27159 | 10.51624 | 0.000283 | 0.0000946 | 0.3022 | 0.158947 | 0.010547 | 7908 | 3345.0625 |
| JK | 10 | 52.27673 | 10.56863 | 0.000285 | 0.0000742 | 0.183178 | 0 | 0.131515 | 3110 | 3982.68 |
| KZ | 10 | 52.25645 | 10.5412 | 0.000275 | 0.0001153 | 0.336563 | 0.149928 | 0.008418 | 0 | 34403.24 |
| MB | 10 | 52.25735 | 10.52904 | 0.000311 | 0.0001328 | 0.292515 | 0.183041 | 0.009128 | 4424 | 1134 |
| MD | 10 | 52.25913 | 10.46925 | 0.000254 | 0.0001602 | 0.043503 | 0.562089 | 0.079856 | 10244 | 11553.475 |
| MS | 10 | 52.22651 | 10.52247 | 0.000258 | 0.0000887 | 0.165004 | 0.305178 | 0.035704 | 6242 | 28980 |
| RK | 10 | 52.22364 | 10.504 | 0.00026 | 0.0000851 | 0.168158 | 0.274267 | 0.011084 | 5070 | 21157.5 |
| TS | 11 | 52.29577 | 10.53531 | 0.000314 | 0.0001206 | 0.094809 | 0.222298 | 0.244333 | 4344 | 5821.125 |
| TU | 10 | 52.28519 | 10.54078 | 0.000271 | 0.0001092 | 0.218623 | 0.133631 | 0.031798 | 0 | 38214.75 |
| TW | 10 | 52.2668 | 10.53224 | 0.000261 | 0.0001167 | 0.39067 | 0.165808 | 0 | 3532 | 19311.125 |

# **Table S3.** Summary of genome assembly quality and statistics for *A. florea* and *A. vaga*, including completeness assessment results, length statistics and composition.

| Species | Busco completeness assessment results | N50 | Mean sequence length (nt) | Median sequence length (nt) | Genome size (bp) |
| --- | --- | --- | --- | --- | --- |
|  |  |  |  |  |  |
| *Andrena florea* |  |  |  |  |  |
| Wengan | 91.17% | 40642 | 25586 | 15308 | 251989019 |
| Spades | 90.12% | 37286 | 4913 | 355 | 281062520 |
|  |  |  |  |  |  |
| *Andrena vaga* |  |  |  |  |  |
| Wengan | 95.24% | 260208 | 81905 | 24349 | 288782998 |
| Spades | 86.56% | 6567 | 433 | 97 | 401540194 |

# **Table S4.** *Andrena florea* sequencing depth, genome coverage and number of read pairs per individual.

| File | Population | Sequencing depth | Coverage (%) | Number of read pairs |
| --- | --- | --- | --- | --- |
| AF-M2_good.rmd | M | 7.99 | 99 | 13870756 |
| PT_AF_B1_S74.rmd | B | 9.41 | 99 | 11551324 |
| PT_AF_B2_S81.rmd | B | 13.08 | 99 | 15513020 |
| PT_AF_B3_S2.rmd | B | 14.90 | 99 | 18149516 |
| PT_AF_B4_S36.rmd | B | 16.82 | 99 | 21149047 |
| PT_AF_B5_S18.rmd | B | 12.67 | 99 | 14902365 |
| PT_AF_B6_S26.rmd | B | 12.20 | 99 | 14761929 |
| PT_AF_B7_S44.rmd | B | 43.48 | 99 | 57500000 |
| PT_AF_EF1_S3.rmd | EF | 17.63 | 99 | 20374223 |
| PT_AF_EF2_S11.rmd | EF | 16.29 | 99 | 19710442 |
| PT_AF_EF3_S19.rmd | EF | 12.81 | 99 | 14821663 |
| PT_AF_EF4_S27.rmd | EF | 11.34 | 99 | 13759517 |
| PT_AF_EF5_S56.rmd | EF | 33.57 | 99 | 42606364 |
| PT_AF_EF6_S41.rmd | EF | 10.76 | 99 | 12711516 |
| PT_AF_EF7_S48.rmd | EF | 12.76 | 99 | 15417380 |
| PT_AF_F1_S40.rmd | F | 13.94 | 99 | 16152307 |
| PT_AF_F2_S47.rmd | F | 13.85 | 99 | 16864901 |
| PT_AF_F3_S54.rmd | F | 8.57 | 99 | 9659966 |
| PT_AF_F4_S61.rmd | F | 8.54 | 99 | 9770341 |
| PT_AF_F5_S102.rmd | F | 13.02 | 99 | 16504826 |
| PT_AF_F6_S75.rmd | F | 14.34 | 99 | 16584264 |
| PT_AF_F7_S35.rmd | F | 24.77 | 99 | 32851273 |
| PT_AF_GA1_S36.rmd | GA | 8.83 | 99 | 10546089 |
| PT_AF_GA2_S40.rmd | GA | 29.11 | 99 | 41141323 |
| PT_AF_GA3_S50.rmd | GA | 9.32 | 99 | 11321773 |
| PT_AF_GA4_S57.rmd | GA | 9.44 | 99 | 11057719 |
| PT_AF_GA5_S99.rmd | GA | 13.13 | 99 | 16914615 |
| PT_AF_GA6_S71.rmd | GA | 16.15 | 99 | 21245059 |
| PT_AF_GA7_S106.rmd | GA | 11.62 | 99 | 16102009 |
| PT_AF_JKI1_S38.rmd | JKI | 29.67 | 99 | 39230540 |
| PT_AF_JKI2_S62.rmd | JKI | 20.56 | 99 | 25379629 |
| PT_AF_JKI3_S69.rmd | JKI | 17.64 | 99 | 21454169 |
| PT_AF_JKI4_S76.rmd | JKI | 13.37 | 99 | 15813574 |
| PT_AF_JKI5_S83.rmd | JKI | 17.85 | 99 | 20622115 |
| PT_AF_JKI6_S4.rmd | JKI | 15.74 | 99 | 18195780 |
| PT_AF_JKI7_S12.rmd | JKI | 10.74 | 99 | 15754363 |
| PT_AF_M1_S39.rmd | M | 28.01 | 99 | 37904909 |
| PT_AF_M3_S31.rmd | M | 10.73 | 99 | 13240762 |
| PT_AF_M4_S41.rmd | M | 33.33 | 99 | 48820614 |
| PT_AF_M5_S65.rmd | M | 14.61 | 99 | 17964070 |
| PT_AF_M6_S84.rmd | M | 21.74 | 99 | 27679395 |
| PT_AF_M7_S59.rmd | M | 15.25 | 99 | 17865430 |
| PT_AF_S1_S88.rmd | S | 23.76 | 99 | 32075109 |
| PT_AF_S2__S94.rmd | S | 17.54 | 99 | 24402227 |
| PT_AF_S3_R_S65.rmd | S | 11.95 | 99 | 14465397 |
| PT_AF_S4_S72.rmd | S | 11.58 | 99 | 13999842 |
| PT_AF_S5_S79.rmd | S | 15.83 | 99 | 18476275 |
| PT_AF_S6_S86.rmd | S | 11.82 | 99 | 14840685 |
| PT_AF_S7_S30.rmd | S | 17.24 | 99 | 21868808 |
| PT_AF_SH1_S43.rmd | SH | 20.83 | 99 | 29485466 |
| PT_AF_SH2_S98.rmd | SH | 12.75 | 99 | 16219945 |
| PT_AF_SH3_S84.rmd | SH | 13.84 | 99 | 15976772 |
| PT_AF_SH4_S33.rmd | SH | 15.59 | 99 | 20334829 |
| PT_AF_SH5_S13.rmd | SH | 10.39 | 99 | 12158819 |
| PT_AF_SH6_S21.rmd | SH | 9.04 | 99 | 10388944 |
| PT_AF_SH7_S29.rmd | SH | 9.49 | 99 | 11060798 |
| PT_AF_TU1_S20.rmd | TU | 15.72 | 99 | 18266226 |
| PT_AF_TU2_S28.rmd | TU | 9.13 | 99 | 10743401 |
| PT_AF_TU3_S89.rmd | TU | 17.24 | 99 | 21329436 |
| PT_AF_TU4_S42.rmd | TU | 15.45 | 99 | 18291735 |
| PT_AF_TU5_S80.rmd | TU | 21.45 | 99 | 28809680 |
| PT_AF_TU6_S56.rmd | TU | 10.65 | 99 | 12634347 |
| PT_AF_TU7_S63.rmd | TU | 17.34 | 99 | 20422380 |
| PT_AF_VE1_S85.rmd | VE | 10.12 | 99 | 11805466 |
| PT_AF_VE2_S35.rmd | VE | 11.78 | 99 | 14473706 |
| PT_AF_VE3_S14.rmd | VE | 12.99 | 99 | 15263359 |
| PT_AF_VE4_S22.rmd | VE | 11.79 | 99 | 14167118 |
| PT_AF_VE5_S30.rmd | VE | 12.89 | 99 | 17634766 |
| PT_AF_VE6_S37.rmd | VE | 8.98 | 99 | 10370376 |
| PT_AF_VE8_S44.rmd | VE | 16.53 | 99 | 19984820 |

# **Table S5.** *Andrena vaga* sequencing depth, genome coverage and number of read pairs per individual.

| Individual ID | Population | Sequencing depth | Coverage (%) | Number of read pairs |
| --- | --- | --- | --- | --- |
| AV0064_good.rmd | IW | 9.91 | 99 | 16294596 |
| AV0201_good.rmd | MB | 9.25 | 98 | 15790583 |
| AV0248_good.rmd | ES | 9.53 | 99 | 14464342 |
| AV0356_good.rmd | TS | 8.78 | 98 | 14537865 |
| AV0580_good.rmd | ES | 9.5 | 99 | 13952488 |
| LB_02_S21.rmd | TU | 22.11 | 99 | 22689173 |
| LB_04_S29.rmd | TU | 24.72 | 99 | 26417764 |
| LB_09_S37.rmd | TU | 24.01 | 99 | 26398242 |
| LB_10_S45.rmd | RK | 32.37 | 99 | 34148024 |
| LB_103_S74.rmd | TU | 23.88 | 99 | 25055461 |
| LB_105_S82.rmd | RK | 20.33 | 99 | 21365882 |
| LB_108_S90.rmd | RK | 13.8 | 99 | 13830268 |
| LB_109_S3.rmd | RK | 19.01 | 99 | 19914815 |
| LB_11_S53.rmd | TU | 25.13 | 99 | 26126222 |
| LB_155_S81.rmd | MB | 19.83 | 99 | 21132908 |
| LB_157_S2.rmd | MB | 14.4 | 99 | 14833520 |
| LB_158_S10.rmd | MB | 24.04 | 99 | 25264940 |
| LB_160_S18.rmd | MB | 18.42 | 99 | 18763599 |
| LB_167_S36.rmd | MD | 30.19 | 99 | 31968356 |
| LB_170_S44.rmd | MD | 17.19 | 99 | 14833520 |
| LB_171_S52.rmd | MD | 31.99 | 99 | 25264940 |
| LB_174_S60.rmd | MD | 24.59 | 99 | 18763599 |
| LB_175_S68.rmd | MD | 22.91 | 99 | 31968356 |
| LB_214_S64.rmd | ES | 15.25 | 99 | 15590753 |
| LB_222_S72.rmd | ES | 7.11 | 96 | 7061436 |
| LB_226_S80.rmd | ES | 6.22 | 94 | 6063033 |
| LB_229_S88.rmd | ES | 15.74 | 99 | 16024157 |
| LB_249_S56.rmd | ES | 16.93 | 99 | 17255255 |
| LB_297_S86.rmd | KZ | 14.14 | 99 | 14509994 |
| LB_298_S94.rmd | KZ | 16.5 | 99 | 16578665 |
| LB_300_S7.rmd | KZ | 17.33 | 99 | 18135510 |
| LB_302_S15.rmd | KZ | 23.07 | 99 | 23116750 |
| LB_303_S23.rmd | KZ | 26.1 | 99 | 26912681 |
| LB_345_S91.rmd | TS | 15.81 | 99 | 15915665 |
| LB_347_S4.rmd | TS | 6.41 | 94 | 6265673 |
| LB_353_S12.rmd | TS | 19.47 | 99 | 19408662 |
| LB_354_S20.rmd | TS | 19.59 | 99 | 19828235 |
| LB_355_S28.rmd | TS | 24.09 | 99 | 25790641 |
| LB_373_S1.rmd | JK | 14.31 | 99 | 13756995 |
| LB_377_S9.rmd | JK | 20.21 | 99 | 19716317 |
| LB_379_S17.rmd | JK | 17.37 | 99 | 18281021 |
| LB_380_S25.rmd | JK | 7.91 | 97 | 8124440 |
| LB_392_S33.rmd | JK | 15.97 | 99 | 16470446 |
| LB_45_S71.rmd | IW | 21.66 | 99 | 22407768 |
| LB_507_S16.rmd | IW | 17.48 | 99 | 17254473 |
| LB_508_S24.rmd | IW | 25.08 | 99 | 26086720 |
| LB_509_S32.rmd | IW | 16.01 | 99 | 15452141 |
| LB_51_S79.rmd | IW | 19.07 | 99 | 19595801 |
| LB_510_S40.rmd | IW | 17.44 | 99 | 17058974 |
| LB_511_S48.rmd | IW | 16.77 | 99 | 17270508 |
| LB_535_S41.rmd | JK | 25.49 | 99 | 27209991 |
| LB_536_S49.rmd | JK | 15.5 | 99 | 16363632 |
| LB_537_S57.rmd | JK | 16.71 | 99 | 16873198 |
| LB_538_S65.rmd | JK | 25.74 | 99 | 27050598 |
| LB_539_S73.rmd | JK | 14.5 | 99 | 14832076 |
| LB_554_S11.rmd | RK | 21.15 | 99 | 20663549 |
| LB_555_S19.rmd | RK | 29.5 | 99 | 29387973 |
| LB_556_S27.rmd | RK | 19.51 | 99 | 19915676 |
| LB_557_S35.rmd | RK | 28.16 | 99 | 28850130 |
| LB_558_S43.rmd | RK | 18.51 | 99 | 18497045 |
| LB_559_S31.rmd | KZ | 18.41 | 99 | 17900235 |
| LB_560_S39.rmd | KZ | 8.77 | 98 | 8522815 |
| LB_561_S47.rmd | KZ | 23.77 | 99 | 24126661 |
| LB_562_S55.rmd | KZ | 21.03 | 99 | 21016749 |
| LB_563_S63.rmd | KZ | 18.93 | 99 | 19100960 |
| LB_575_S97.rmd | ES | 10.23 | 99 | 10110542 |
| LB_576_S98.rmd | ES | 12.35 | 99 | 12294819 |
| LB_577_S99.rmd | ES | 7.72 | 97 | 7625237 |
| LB_578_S100.rmd | ES | 5.36 | 90 | 5299577 |
| LB_579_S101.rmd | ES | 8.97 | 98 | 9029598 |
| LB_58_S87.rmd | TS | 10.51 | 98 | 10910317 |
| LB_585_S51.rmd | IW | 22.5 | 99 | 22208734 |
| LB_587_S59.rmd | TS | 28.51 | 99 | 28675597 |
| LB_588_S67.rmd | TS | 19.26 | 99 | 18983830 |
| LB_589_S75.rmd | TS | 10.4 | 98 | 10325375 |
| LB_590_S83.rmd | TS | 23.9 | 99 | 23810077 |
| LB_596_S26.rmd | MB | 26.4 | 99 | 27233965 |
| LB_597_S34.rmd | MB | 30.73 | 99 | 31246442 |
| LB_598_S42.rmd | MB | 11.08 | 99 | 11224643 |
| LB_599_S50.rmd | MB | 24.34 | 99 | 24838460 |
| LB_60_S95.rmd | MB | 5.69 | 91 | 5563394 |
| LB_600_S58.rmd | IW | 9.11 | 98 | 9107458 |
| LB_610_S61.rmd | TU | 29.11 | 99 | 30111524 |
| LB_611_S69.rmd | TU | 7.76 | 96 | 7913117 |
| LB_612_S77.rmd | TU | 22.92 | 99 | 22670766 |
| LB_613_S85.rmd | TU | 20.86 | 99 | 20366319 |
| LB_614_S93.rmd | TU | 15.22 | 99 | 15109237 |
| LB_62_S8.rmd | TW | 16.33 | 99 | 17129472 |
| LB_621_S46.rmd | IW | 20.29 | 99 | 20178259 |
| LB_622_S54.rmd | TW | 20.33 | 99 | 19888674 |
| LB_623_S62.rmd | TW | 23.31 | 99 | 22921895 |
| LB_624_S70.rmd | TW | 27.89 | 99 | 27991613 |
| LB_628_S78.rmd | TW | 19.98 | 99 | 19936519 |
| LB_634_S76.rmd | MD | 25.17 | 99 | 25765644 |
| LB_635_S84.rmd | MD | 20.8 | 99 | 21712262 |
| LB_636_S92.rmd | MD | 17.59 | 99 | 17957364 |
| LB_637_S5.rmd | MD | 19.59 | 99 | 19635519 |
| LB_638_S13.rmd | MD | 25.41 | 99 | 26168713 |
| LB_77_S6.rmd | TW | 16.3 | 99 | 16760778 |
| LB_78_S14.rmd | TW | 22.06 | 99 | 22121350 |
| LB_79_S22.rmd | TW | 20.16 | 99 | 20959217 |
| LB_81_S30.rmd | TW | 20.98 | 99 | 21831492 |
| LB_83_S38.rmd | TW | 23.75 | 99 | 25199879 |
| LB_96_S66.rmd | RK | 15.23 | 99 | 15544114 |
| PT_AV0250_S1.rmd | MS | 12.68 | 99 | 13087448 |
| PT_AV0251_S9.rmd | MS | 18.32 | 99 | 18535950 |
| PT_AV0253_S17.rmd | MS | 21.41 | 99 | 22719417 |
| PT_AV0254_S25.rmd | MS | 19.54 | 99 | 20428538 |
| PT_AV0255_S32.rmd | MS | 19.67 | 99 | 20434646 |
| PT_AV0522_S39.rmd | MS | 15.4 | 99 | 15681770 |
| PT_AV0523_S46.rmd | MS | 16.33 | 99 | 16495429 |
| PT_AV0524_S53.rmd | MS | 21 | 99 | 21171788 |
| PT_AV0525_S60.rmd | MS | 12.08 | 99 | 12638000 |
| PT_AV0526_S33.rmd | MS | 38.77 | 99 | 44967725 |

# **Table S6**. *Andrena florea* estimated pairwise *F*_ST_ between all population pairs (above diagonal). P-values from 1000 bootstraps (below diagonal).

|  | B | EF | F | GA | JK | M | S | SH | TU | VE |
| --- | --- | --- | --- | --- | --- | --- | --- | --- | --- | --- |
| B | NA | 0.07273 | 0.008196 | 0.012564 | 0.062291 | 0.031858 | 0.052566 | 0.057858 | 0.058034 | 0.028867 |
| EF | 0 | NA | 0.058911 | 0.036343 | 0.003641 | 0.020328 | 0.010609 | 0.008777 | 0.007743 | 0.057838 |
| F | 0 | 0 | NA | 0.003784 | 0.048788 | 0.017378 | 0.039061 | 0.043727 | 0.041699 | 0.017629 |
| GA | 0 | 0 | 0 | NA | 0.027011 | 0.002106 | 0.018412 | 0.018467 | 0.024619 | 0.011543 |
| JK | 0 | 0 | 0 | 0 | NA | 0.015807 | 0.00402 | 0.00566 | 0 | 0.048516 |
| M | 0 | 0 | 0 | 0 | 0 | NA | 0.008193 | 0.012909 | 0.015834 | 0.014303 |
| S | 0 | 0 | 0 | 0 | 0 | 0 | NA | 0.002343 | 0.010928 | 0.03374 |
| SH | 0 | 0 | 0 | 0 | 0 | 0 | 0 | NA | 0.007855 | 0.039898 |
| TU | 0 | 0 | 0 | 0 | 1 | 0 | 0 | 0 | NA | 0.04182 |
| VE | 0 | 0 | 0 | 0 | 0 | 0 | 0 | 0 | 0 | NA |

# **Table S7.** *Andrena vaga* estimated pairwise *F*_ST_ between all location pairs (above diagonal). P-values from 1000 bootstraps (below diagonal).

|  | ES | IW | JK | KZ | MB | MD | MS | RK | TS | TU | TW |
| --- | --- | --- | --- | --- | --- | --- | --- | --- | --- | --- | --- |
| ES | NA | 0.004084 | 0.004213 | 0.006512 | 0.004401 | 0.006135 | 0.008388 | 0.007291 | 0.004705 | 0.003817 | 0.004384 |
| IW | 0 | NA | 0.001565 | 0.003467 | 0.000941 | 0.00179 | 0.005142 | 0.002514 | 0.002427 | 0.001302 | 0.000801 |
| JK | 0 | 0 | NA | 0.003441 | 0.001124 | 0.00188 | 0.004627 | 0.002405 | 0.002893 | 0.001024 | 0.001388 |
| KZ | 0 | 0 | 0 | NA | 0.002661 | 0.003161 | 0.006511 | 0.004248 | 0.005558 | 0.004528 | 0.003828 |
| MB | 0 | 0 | 0 | 0 | NA | 0.001269 | 0.004153 | 0.002208 | 0.003541 | 0.002154 | 0.001605 |
| MD | 0 | 0 | 0 | 0 | 0 | NA | 0.004401 | 0.001732 | 0.004015 | 0.001049 | 0.0015 |
| MS | 0 | 0 | 0 | 0 | 0 | 0 | NA | 0.003564 | 0.007172 | 0.005581 | 0.004842 |
| RK | 0 | 0 | 0 | 0 | 0 | 0 | 0 | NA | 0.005369 | 0.003029 | 0.001879 |
| TS | 0 | 0 | 0 | 0 | 0 | 0 | 0 | 0 | NA | 0.002538 | 0.00359 |
| TU | 0 | 0 | 0 | 0 | 0 | 0 | 0 | 0 | 0 | NA | 0.0012 |
| TW | 0 | 0 | 0 | 0 | 0 | 0 | 0 | 0 | 0 | 0 | NA |

# **List of supplementary data tables in an Excel file**

| **Table S8.** Urban intensity-associated SNPs detected using LFMM in *Andrena florea*. The contig and SNP position are reported. |
| --- |
| **Table S9.** Urban intensity-associated SNPs detected using LFMM in *Andrena vaga*. The contig and SNP position are reported. |
| **Table S10.** Urban intensity-associated SNPs detected using Gradient Forest in *Andrena florea*. The contig and SNP position are reported. |
| **Table S11.** Urban intensity-associated SNPs detected using Gradient Forest in *Andrena vaga*. The contig and SNP position are reported. |
| **Table S12.** Urban intensity-associated SNPs detected using LFMM and Gradient Forest (LFMM ∩ GF) in *Andrena florea*. The contig and SNP position are reported. SNPs highlighted in the table correspond to loci that also overlap regions identified as putative selective sweeps based on nSL analyses. |
| **Table S13.** Urban intensity-associated SNPs detected using LFMM and Gradient Forest (LFMM ∩ GF) in *Andrena vaga*. The contig and SNP position are reported. SNPs highlighted in the table correspond to loci that also overlap regions identified as putative selective sweeps based on nSL analyses. |
| **Table S14.** Annotation of the genes identified to be associated with urban intensity using LFMM ∩ GF in *Andrena florea*. The contig, SNP position, gene and protein are reported. SNPs highlighted in the table correspond to loci that also overlap regions identified as putative selective sweeps based on nSL analyses. |
| **Table S15.** Annotation of the genes identified to be associated with urban intensity using LFMM ∩ GF in *Andrena vaga*. The contig, SNP position, gene and protein are reported. SNPs highlighted in the table correspond to loci that also overlap regions identified as putative selective sweeps based on nSL analyses. |
| **Table S16.** Gene ontology enrichment analysis of LFMM ∩ GF urban intensity associated SNPs in *Andrena florea*. GO.ID: Identifier for the Gene Ontology term. Term: Description or name of the GO term associated with the GO.ID. Annotated: The total number of genes in the background set that are annotated with the specific GO term. Significant: Number of genes in our list of interest that are annotated with the specific GO term. Expected: This is the number of genes one would expect to be annotated with the specific GO term in our list of interest if there were no enrichment. It is calculated based on the proportion of genes annotated with the GO term in the background set. P-value: Statistical significance of the enrichment of the GO term in our list of interest. |
| **Table S17.** Gene ontology enrichment analysis of LFMM ∩ GF urban intensity associated SNPs in *Andrena vaga*. GO.ID: Identifier for the Gene Ontology term. Term: Description or name of the GO term associated with the GO.ID. Annotated: The total number of genes in the background set that are annotated with the specific GO term. Significant: Number of genes in our list of interest that are annotated with the specific GO term. Expected: This is the number of genes one would expect to be annotated with the specific GO term in our list of interest if there were no enrichment. It is calculated based on the proportion of genes annotated with the GO term in the background set. P-value: Statistical significance of the enrichment of the GO term in our list of interest. |
| **Table S18.** Gene ontology enrichment analysis of LFMM ∩ GF ∩ nSL SNPs in *Andrena florea*. GO.ID: Identifier for the Gene Ontology term. Term: Description or name of the GO term associated with the GO.ID. Annotated: The total number of genes in the background set that are annotated with the specific GO term. Significant: Number of genes in our list of interest that are annotated with the specific GO term. Expected: This is the number of genes one would expect to be annotated with the specific GO term in our list of interest if there were no enrichment. It is calculated based on the proportion of genes annotated with the GO term in the background set. P-value: Statistical significance of the enrichment of the GO term in our list of interest. |
| **Table S19.** Gene ontology enrichment analysis of LFMM ∩ GF ∩ nSL SNPs in *Andrena vaga*. GO.ID: Identifier for the Gene Ontology term. Term: Description or name of the GO term associated with the GO.ID. Annotated: The total number of genes in the background set that are annotated with the specific GO term. Significant: Number of genes in our list of interest that are annotated with the specific GO term. Expected: This is the number of genes one would expect to be annotated with the specific GO term in our list of interest if there were no enrichment. It is calculated based on the proportion of genes annotated with the GO term in the background set. P-value: Statistical significance of the enrichment of the GO term in our list of interest. |
| Table S20. Urban intensity-associated sequence orthologues between *Andrena florea* and *Andrena vaga*. The species, contig ID, SNP position, and corresponding orthologue group are reported. |
| **Table S21.** Annotation of the genes identified to be associated with urban intensity in both *Andrena florea* and *Andrena vaga*. The gene and protein are reported. |
| **Table S22.** Gene ontology enrichment analysis of LFMM ∩ GF urban intensity associated SNPs in *Andrena florea* and *Andrena vaga*. GO.ID: Identifier for the Gene Ontology term. Term: Description or name of the GO term associated with the GO.ID. Annotated: The total number of genes in the background set that are annotated with the specific GO term. Significant: Number of genes in our list of interest that are annotated with the specific GO term. Expected: This is the number of genes one would expect to be annotated with the specific GO term in our list of interest if there were no enrichment. It is calculated based on the proportion of genes annotated with the GO term in the background set. P-value: Statistical significance of the enrichment of the GO term in our list of interest. |

# **References**

Astashyn, A., Tvedte, E. S., Sweeney, D., Sapojnikov, V., Bouk, N., Joukov, V., Mozes, E., Strope, P. K., Sylla, P. M., Wagner, L., Bidwell, S. L., Brown, L. C., Clark, K., Davis, E. W., Smith-White, B., Hlavina, W., Pruitt, K. D., Schneider, V. A., & Murphy, T. D. (2024). Rapid and sensitive detection of genome contamination at scale with FCS-GX. *Genome Biology*, *25*(1), 60. https://doi.org/10.1186/s13059-024-03198-7

Di Genova, A., Buena-Atienza, E., Ossowski, S., & Sagot, M.-F. (2021). Efficient hybrid de novo assembly of human genomes with WENGAN. *Nature Biotechnology*, *39*(4), 422–430. https://doi.org/10.1038/s41587-020-00747-w

Hu, J., Fan, J., Sun, Z., & Liu, S. (2020). NextPolish: A fast and efficient genome polishing tool for long-read assembly. *Bioinformatics*, *36*(7), 2253–2255. https://doi.org/10.1093/bioinformatics/btz891

Nishimura, O., Hara, Y., & Kuraku, S. (2017). gVolante for standardizing completeness assessment of genome and transcriptome assemblies. *Bioinformatics (Oxford, England)*, *33*(22), 3635–3637. https://doi.org/10.1093/bioinformatics/btx445

Prjibelski, A., Antipov, D., Meleshko, D., Lapidus, A., & Korobeynikov, A. (2020). Using SPAdes De Novo Assembler. *Current Protocols in Bioinformatics*, *70*(1), e102. https://doi.org/10.1002/cpbi.102

Seppey, M., Manni, M., & Zdobnov, E. M. (2019). *BUSCO: Assessing Genome Assembly and Annotation Completeness* (pp. 227–245). Humana, New York, NY. https://doi.org/10.1007/978-1-4939-9173-0_14
